# Exploring Romanian Pharmacists’ Migration Readiness Through the Push–Pull–Mooring Framework: A PLS-SEM Study

**DOI:** 10.3390/healthcare14193204

**Published:** 2026-09-28

**Authors:** Alin-Viorel Focșa, Ionuț-Iulian Lungu, Maria Drăgan, Magdalena Bîrsan, Ioana-Cezara Caba, Ruxandra-Teodora Stan, Maria-Manuela Apostol, Pompei Mititean, Cătălina Daniela Stan

**Affiliations:** 1 Grigore T. Popa University of Medicine and Pharmacy Iasi, 700115 Iași, Romania; alin-viorel.focsa@umfiasi.ro (A.-V.F.); maria.wolszleger@umfiasi.ro (M.D.); magdalena.birsan@umfiasi.ro (M.B.); ioana-cezara.caba@umfiasi.ro (I.-C.C.); ruxandra-teodora.stan@umfiasi.ro (R.-T.S.); maria_manuela.apostol@umfiasi.ro (M.-M.A.); catalina.stan@umfiasi.ro (C.D.S.); 2 High School “Mircea cel Bătrân”, 012206 Bucharest, Romania; 3 National School of Political and Administrative Studies, 012104 Bucharest, Romania

**Keywords:** pharmacist migration, migration readiness, health workforce, workforce retention, evidence-based public health, health workforce planning, Push–Pull–Mooring, PLS-SEM, predictive assessment

## Abstract

**Background:** The international migration of pharmaceutical professionals has implications for the availability of human resources and the sustainability of pharmaceutical services; however, the determinants of pharmacists’ readiness for cross-border mobility remain insufficiently examined. This study examines the associations of professional factors, the importance assigned to migration-related benefits, and relocation-related barriers with Migration Readiness within the Push–Pull–Mooring (PPM) theoretical framework. **Methods:** Data were collected via an online questionnaire administered to a sample of 542 professionally active pharmacists in Romania and were analyzed using Partial Least Squares Structural Equation Modeling (PLS-SEM) with SmartPLS 4. The findings indicate that dissatisfaction with working conditions and remuneration is positively associated with both the Importance of Migration Benefits and Migration Readiness. Greater importance assigned to potential economic, professional, and personal migration-related benefits is, in turn, positively associated with Migration Readiness. **Results:** Among the mooring factors, Migration Mooring Barriers exhibit the strongest direct association with Migration Readiness, with a negative direction of the relationship; within this construct, family responsibilities and uncertainty regarding adjustment displayed the largest positive relative weights within the formative composite. By contrast, perceived institutional and professional deficits do not display significant associations with either perceived migration benefits or readiness to migrate. In addition, the internal cross-validated predictive assessment yielded positive Q^2^ predict values for both endogenous constructs (Importance of Migration Benefits, IMB = 0.210; Migration Readiness, MR = 0.193). CVPAT indicated significantly lower predictive loss for the PLS-SEM model than for the indicator-mean and linear-model benchmarks, although the predictive advantage over the linear model was modest. These results support the internal predictive performance of the selected model for questionnaire responses within the analyzed sample. They do not establish the model’s ability to predict subsequent migration or monitor workforce vulnerability over time. The model should therefore be regarded as a candidate framework for future longitudinal and externally validated workforce research. **Conclusions:** The findings provide exploratory support for examining pharmacists’ Migration Readiness within the PPM framework. Because the instrument was author-developed or conceptually adapted from previous literature, measurement evaluation and refinement and structural-model testing were conducted within the same convenience sample, and the convergent validity of the formative composites was not established through redundancy analysis, the results require independent replication. The model should not be regarded as a validated workforce-monitoring, individual-prediction, or decision-support instrument.

## 1. Introduction

Migration of health professionals can affect the sustainability of health systems by increasing staff shortages, uneven distribution of skills, and reducing continuity of services. Differences between countries in remuneration, working conditions, development opportunities, and quality of life lead some professionals to consider international mobility as a career option [1,2].

The literature has primarily focused on doctors and nurses, while the migration of pharmacists has been less analysed. This gap is relevant because pharmacists contribute to the safe use of medicines, patient counselling, treatment monitoring, and the provision of clinical, preventive, and public health services. Existing studies indicate that pharmacist mobility is associated with economic, professional, and personal factors. Insufficient remuneration, unfavorable working conditions, limited development prospects and low recognition of the profession can favor the evaluation of external alternatives [3,4]. Also, job satisfaction, occupational stress, organizational climate and work–life balance are associated with the intention to leave or emigrate [5,6,7,8].

However, job dissatisfaction does not automatically lead to migration. External advantages may be evaluated favorably, but family responsibilities, relocation costs, language difficulties and uncertainty of adaptation can limit mobility. Migration should therefore be analyzed as a comparative process between the current professional situation, the benefits attributed to the destination and the feasibility of relocation.

Romania is a relevant context, as membership in the European Union facilitates pharmacists’ access to other labor markets. Previous research has highlighted external mobility, but also dissatisfaction with remuneration, professional recognition, institutional support, and development opportunities [9,10,11,12]. However, the mechanisms through which these perceptions are associated with Migration Readiness remain insufficiently investigated.

Existing research on pharmacists has primarily examined individual economic and professional determinants of migration, direct associations with migration intention, or related outcomes such as job turnover and professional retention [2,3,4,5,6,7]. Comparatively less attention has been given to an integrated framework that simultaneously distinguishes immediate dissatisfaction with work and remuneration, broader assessments of institutional and professional conditions, the importance assigned to potential migration-related benefits, and the barriers that may constrain mobility. Previous pharmacist-focused studies have also rarely examined whether push-related professional dissatisfaction is associated with Migration Readiness both directly and through the importance assigned to potential migration benefits. The research gap is therefore not limited to the smaller number of studies concerning pharmacists; it also concerns the limited integration of push, pull, and mooring dimensions and the limited examination of specific indirect associations within this professional group.

The contribution of the present study is threefold. Theoretically, it applies the PPM framework by distinguishing immediate work-and-pay dissatisfaction from broader institutional and professional assessments and by examining direct and specific indirect associations through Importance of Migration Benefits. Contextually, it examines these relationships among professionally active pharmacists in Romania, while explicitly recognizing that the convenience sample does not support population-level generalization. Methodologically, it evaluates a provisional author-developed or conceptually adapted measurement specification and assesses the robustness of the Migration Readiness operationalization through alternative analyses. Its practical contribution is limited to identifying domains for future retention and workforce research; the study does not validate an operational monitoring or decision-support instrument.

From a health-systems perspective, professional migration may affect the availability and stability of human resources. Survey-based measures of migration readiness and associated factors could potentially complement administrative workforce indicators by capturing perceptions that are not directly represented in staffing numbers, vacancy rates, or geographical-distribution statistics. However, before such measures can be used for workforce monitoring, their temporal stability, external validity, comparability across professional groups, and association with subsequent migration behavior must be established.

To address this gap, the research uses the Push–Pull–Mooring model, based on [13] migration theory and the concept of moorings formulated by [14]. The model allows for the simultaneous analysis of factors that move the individual away from the current situation, the advantages attributed to the destination and the barriers that limit change [15,16].

The push component is represented by Work and Pay Push Factors, which capture dissatisfaction with working conditions and remuneration, and by Perceived Institutional and Professional Deficit, which reflects institutional support, professional recognition, and development prospects. The pull component is operationalized as Importance of Migration Benefits, while the mooring component is represented by Migration Mooring Barriers. Migration Readiness is used as a provisional umbrella construct describing a pre-behavioral orientation toward considering professional migration, regarding it as a feasible career option, and potentially progressing toward preparatory action. It differs from migration aspiration, which may express a general desire; from a specific migration intention, which normally refers to a stated plan to migrate; and from actual migration behavior, which requires observed relocation. The study does not claim that Migration Readiness represents a universally established and distinct stage of migration. Its proposed value lies in integrating consideration, perceived feasibility, intended timing, and initial preparation within the present exploratory framework, a specification that requires further independent validation.

The objective of this study was to examine factors associated with pharmacists’ Migration Readiness in Romania within the Push–Pull–Mooring framework. The study tested the hypothesized relationships among push, pull, and mooring factors, examined the specific indirect associations through Importance of Migration Benefits, and assessed the internal cross-validated predictive performance of the selected model. It did not test the prediction of actual subsequent migration or validate the instrument as a workforce-monitoring system.

The contribution of the study is primarily theoretical and exploratory. It extends the application of the Push–Pull–Mooring framework to the pharmaceutical workforce by distinguishing immediate dissatisfaction with work and remuneration from broader institutional assessments and by examining the indirect association transmitted through Importance of Migration Benefits. The PLSpredict and CVPAT analyses provide an internal assessment of the selected model’s ability to predict held-out questionnaire responses. This predictive assessment represents an initial methodological step and should not be interpreted as validation of a workforce-monitoring or decision-support instrument.

## 2. Literature Review and Hypothesis Development

### 2.1. Migration of Health Professionals and the Specificity of the Pharmaceutical Profession

Migration of health professionals represents a major challenge for the sustainability of health systems, as it can exacerbate staff shortages, uneven distribution of skills and discontinuity of services provided to the population. Although research has mainly focused on doctors and nurses, pharmacists represent an essential category for the functioning of health services, through their responsibilities regarding the safe use of medicines, patient counselling and treatment monitoring [1,4].

The literature on the mobility of health professionals consistently identifies remuneration, working conditions, career prospects and job satisfaction among the main factors associated with migration or intention to migrate [1]. In the case of pharmacists, the intention to leave is also associated with professional stress, organizational climate, perceived support and career development opportunities [5,7].

Although research dedicated exclusively to pharmacist migration is less numerous, the existing results confirm the importance of economic, professional and social factors. Perceived differences between the professional environment in the country of origin and the opportunities available abroad influence pharmacists’ orientation towards migration [3,4]. At the same time, professional satisfaction and work–life balance are associated with pharmacists’ intention to emigrate [6].

International migration, leaving an employer, and leaving the pharmacy profession represent conceptually distinct outcomes. Accordingly, studies directly examining international migration, migration intention, or emigration intention provide the primary empirical basis for the migration-related hypotheses in this study. Evidence concerning turnover intention, job quitting, and profession leaving is used only as indirect contextual support for understanding professional dissatisfaction, institutional conditions, and retention pressures. Such evidence is not treated as demonstrating an equivalent association with international migration.

Romania represents a relevant context for the analysis of this phenomenon, because free movement within the European Union facilitates pharmacists’ access to labor markets perceived as offering more advantageous salaries, professional conditions and career opportunities. Research conducted on Romanian pharmacists has highlighted dissatisfaction regarding remuneration equity, professional recognition and professional development prospects [10]. In this context, readiness for migration can be understood as the result of comparing the current professional situation with the anticipated benefits of working abroad and the difficulties associated with relocation.

From this perspective, Migration Readiness may be investigated as a potential perceptual correlate of retention pressure. However, the present cross-sectional study does not establish that Migration Readiness precedes or predicts subsequent staff loss. Determining whether this construct can complement traditional workforce indicators requires longitudinal evidence linking survey responses to later migration behavior.

### 2.2. The Push–Pull–Mooring Theoretical Framework

The Push–Pull–Mooring Model (PPM) provides an appropriate framework for explaining the readiness to migrate, as it integrates three categories of factors. Push factors designate the unfavorable aspects of the current situation, pull factors describe the advantages attributed to the destination, and mooring factors include personal, social, and contextual elements that can facilitate or hinder mobility.

The foundation of the model is found in the migration theory developed by Lee [13], according to which the mobility decision is influenced by favorable and unfavorable factors associated with the place of origin and destination, as well as by intervening obstacles. Ref. [14] extended this perspective by introducing the concept of moorings, respectively the personal, social, and cultural anchors that influence the decision to change. Subsequently, the PPM framework has been used to explain mobility and change behaviors [15,16]. Recent research confirms its usefulness in explaining migration intention, highlighting the distinct effects of dissatisfaction, attractiveness of alternatives, switching costs and social norms [17,18,19].

In the present research, push factors are represented by two constructs. Work and Pay Push Factors (WPPF) capture immediate dissatisfaction with working conditions and remuneration, and Perceived Institutional and Professional Deficit (PID) reflects perceptions of institutional support, professional value and development prospects. Pull factors are represented by Importance of Migration Benefits (IMB), and mooring factors by Migration Mooring Barriers (MB). The dependent variable is Migration Readiness (MR), the provisional pre-behavioral orientation toward considering professional migration, regarding it as feasible, and potentially progressing toward preparatory action.

The PPM avoids the simplistic interpretation of migration as an automatic reaction to dissatisfaction. Even if the current professional situation is assessed unfavourably and external opportunities are attractive, mobility may be limited by family responsibilities, relocation costs, language difficulties or uncertainty of adaptation.

The proposed structural model treats the PPM framework as a set of related pathways rather than only as a classification of determinants. WPPF is associated with MR through two theoretically distinct paths. The direct path represents the possibility that dissatisfaction with current working conditions and remuneration is associated with greater readiness to consider migration independently of the importance assigned to specific external benefits. The specific indirect path through IMB represents the possibility that respondents who report greater dissatisfaction also assign greater importance to economic, professional, and personal migration-related benefits, which is in turn associated with Migration Readiness. Given the cross-sectional design, this pathway is interpreted as a specific indirect association and not as a demonstrated temporal process.

MB was specified as a direct predictor of MR because its indicators capture the perceived intensity of linguistic, regulatory, financial, informational, family, and adaptation-related constraints on relocation. The hypothesized coefficient therefore represents the association between the overall barrier composite and Migration Readiness. Mooring factors could also plausibly moderate the WPPF → MR or IMB → MR relationships, such that push pressures or migration-related benefits may display weaker associations with readiness among respondents reporting greater relocation barriers. However, these interaction effects were not prespecified, and the instrument and analytical strategy were not designed to test a moderated PPM. Moderation should therefore be considered an alternative theoretical specification for future confirmatory research rather than introduced as a post hoc extension of the present model.

### 2.3. Work and Pay Push Factors

Remuneration and working conditions are among the most frequent factors associated with international mobility of health professionals. Salary has both an economic function, through its influence on living standards, and a symbolic one, as it can reflect the value attributed to work and professional skills. When income is considered inadequate in relation to training, responsibilities and volume of work, external professional opportunities may become more attractive [1,4].

Working conditions include the volume of work, schedule, available resources, professional autonomy, clarity of roles and work–life balance. In the case of pharmacists, job satisfaction, stress and organizational environment characteristics are associated with the intention to leave or leave the profession [5,7]. Also, job satisfaction and work–life balance influence pharmacists’ intention to emigrate [6]. This turnover-related evidence is used as indirect support for the relevance of working conditions and professional dissatisfaction and is not interpreted as evidence that leaving an employer or profession is equivalent to international migration.

In the proposed model, working conditions and remuneration represent distinct push factors. Dissatisfaction with these dimensions may accentuate the advantages attributed to external opportunities and increase the willingness to migrate.

**H1.**  *Work and Pay Push Factors are positively associated with the Importance of Migration Benefits.*

**H2.**  *Work and Pay Push Factors are positively associated with Migration Readiness.*

### 2.4. Perceived Institutional and Professional Deficit

Professional experience is not solely determined by the conditions at the workplace, but also by how professionals assess the position of their occupation in the health system and society. Support from authorities, employers and professional organizations, recognition from other health professionals, representation of the interests of the profession and the existence of clear development paths can influence the perception of the viability of a long-term career.

In the case of Australian pharmacists, ref. [20] identified dissatisfaction with the professional environment, lack of career development opportunities and insufficient use of acquired knowledge and skills among the main reasons for leaving the profession. Similar results were reported in New Zealand by [21], who highlighted the lack of recognition of the profession by authorities, the public and other health professionals, insufficient professional representation, the absence of promotion paths and limited access to clinical roles. These findings indicate that intention to leave is not only associated with economic rewards, but also with the perception that the profession does not offer the anticipated status, autonomy, and development.

Evidence summarized by [22] confirms that pharmacists’ intention to leave their job or profession is influenced by organizational and professional factors, such as promotion opportunities, managerial support, recognition, and job satisfaction. Specifically, ref. [23] shows that perceived organizational support, expressed through emotional, instrumental, managerial, and peer support, is negatively associated with pharmacists’ intention to leave primary care.

For pharmacists at the beginning of their careers, the opportunity to progress and acquire new skills is one of the most important determinants of job satisfaction [24]. Similarly, the systematic review by [25], which synthesized evidence from 36 countries, highlights the role of development opportunities, recognition, autonomy, organizational support, and professional policies in the retention of the pharmaceutical workforce.

The perception of an institutional and professional deficit may reflect pharmacists’ concerns regarding insufficient professional representation, underutilization of their competencies, limited organizational support, and restricted opportunities for career development [22,23,26]. Under these circumstances, professional systems in other countries may be assessed as offering a clearer status, greater autonomy, and more consistent opportunities for advancement. However, these assessments concern the general architecture of the profession and the functioning of institutions, and are broader and more stable than direct experiences of salary, workload, or schedule. For this reason, the contribution of Perceived Institutional and Professional Deficit is analyzed separately from Work and Pay Push Factors in explaining Migration Readiness.

**H3.**  *Perceived institutional and professional deficit is positively associated with Importance of Migration Benefits.*

**H4.**  *Perceived Institutional and Professional Deficit is positively associated with Migration Readiness.*

### 2.5. Importance of Migration Benefits

Pull factors represent the economic, professional and personal advantages attributed to working abroad. These may include higher salaries, better working conditions, job stability, professional recognition, access to advanced technologies and resources, training and career development opportunities, and the possibility of achieving a higher standard of living.

The literature on medical staff migration highlights the multidimensional nature of these benefits. The systematic review by [1] shows that career prospects, a favorable work environment, job satisfaction and remuneration are central factors of international mobility. Similarly, ref. [27] highlights the importance of wage differences, the availability of medical equipment, the quality of the professional environment and the stability of the social and political system in the destination countries. In the case of Romania, ref. [28] show that the choice of destination by healthcare professionals is influenced by the level of development of the respective country and by the existence of Romanian professional communities, which can facilitate professional and social integration.

For pharmacists, the professional and economic benefits of migration are supported by both early international research and recent studies. Ref. [4] identifies higher remuneration, employment and training opportunities, better working conditions and a high standard of living as important factors in pharmacist mobility. In Kerala, ref. [3] find that the existence of more professional opportunities abroad and the possibility of obtaining higher salaries significantly increase the likelihood of a high intention to migrate. Similarly ref. [29] show that Italian pharmacists who moved to the UK were particularly attracted by better salaries, clinically oriented professional opportunities and more favorable career development prospects.

In the present study, these domains are not operationalized as respondents’ beliefs that particular benefits are necessarily available abroad. Instead, the construct captures the importance assigned to potential economic, professional, and personal benefits when considering professional migration. Accordingly, Importance of Migration Benefits reflects the salience of these considerations in the migration decision process rather than their expected availability in the destination country.

**H5.**  *Importance of Migration Benefits are positively associated with Migration Readiness.*

### 2.6. Mooring Barriers Associated with Migration

Mooring factors explain why the existence of job dissatisfaction and attractive external opportunities do not always lead to migration. International relocation involves financial resources, the use of a foreign language, the reorganization of family life, and adaptation to a new professional and social environment [13,14].

For healthcare professionals, language skills, cultural integration, social support and adaptation to the professional requirements of the destination country influence the mobility process [30]. For pharmacists, adaptation also involves familiarization with other regulations, professional responsibilities, information systems and organizational practices.

Family responsibilities can function as strong anchors, as the decision to leave affects not only the professional, but also the partner, children or other dependents. Studies on healthcare professionals show that family ties and personal obligations can reduce the willingness to migrate, even when external opportunities are evaluated favorably [31,32].

Therefore, language, relocation costs, family responsibilities and adjustment uncertainty represent distinct barriers that can reduce the willingness to migrate.

**H6.**  *Migration Mooring Barriers are negatively associated with Migration Readiness.*

### 2.7. The Indirect Role of Importance of Migration Benefits

Work and Pay Push Factors may be associated with Migration Readiness both directly and indirectly through the importance assigned to migration-related benefits. When working conditions and remuneration in the current professional environment are evaluated unfavorably, economic, professional, and personal reasons for considering migration may become more salient in the individual decision process. This interpretation concerns the importance assigned to potential migration benefits and does not imply that dissatisfaction changes their objective availability abroad.

In the logic of the Push–Pull–Mooring model, the decision to change involves a comparative assessment of the current situation and the available alternatives. Ref. [15] shows that dissatisfaction with the existing option and the attractiveness of alternatives are distinct components of the change process, both of which contribute to the formation of intention. Similarly, ref. [16] highlights that the factors that push the individual away from the current situation and those that pull them towards an alternative must be analyzed together, since the decision does not depend exclusively on the intensity of dissatisfaction, but also on the existence of a perceived superior option.

This interpretation is compatible with the aspirations–capabilities framework proposed by [33], according to which the aspiration to migrate is formed by relating existing conditions to the opportunities, freedoms and possibilities associated with other contexts. The assessment is not exclusively objective. Ref. [34] emphasizes that the migration decision is influenced by subjective processes, such as imagining life at the destination, anticipating future outcomes and interpreting differences between origin and destination. Therefore, job dissatisfaction can accentuate the contrast between the current situation and the expected benefits of working abroad.

In the field of health, ref. [1] find that insufficient remuneration, unfavorable working environment and limited professional prospects frequently coexist with pull factors such as higher incomes, better professional conditions and development opportunities in the destination countries. Also, ref. [19], applying the PPM to the mobility of medical personnel, show that the attractiveness of alternatives contributes significantly to mobility intention, together with dissatisfaction with the professional situation and perceived barriers.

Consequently, Importance of Migration Benefits may represent an intermediate associative pathway between Work and Pay Push Factors and Migration Readiness. Unsatisfactory salaries and unfavorable working conditions may be associated with higher Migration Readiness both directly and through greater importance assigned to potential economic and professional migration benefits [1,3].

A similar indirect association can be examined for Perceived Institutional and Professional Deficit. Perceptions of low professional recognition, insufficient institutional representation, and limited development opportunities may be associated with the importance assigned to external professional alternatives [20,21,25]. Given the cross-sectional design, this relationship is treated as an indirect association compatible with the PPM framework rather than as evidence of a temporal or causal mechanism.

However, the existing literature frequently analyses push and pull factors as direct or interdependent predictors of mobility intention, without consistently demonstrating a causal sequence between them. Also considering the cross-sectional nature of the present research, the mediation effects are interpreted as indirect associations compatible with the proposed theoretical mechanism, not as evidence of a temporal order or a demonstrated causality.

**H7.**  *Importance* *of Migration Benefits conveys a positive indirect association between Work and Pay Push Factors and Migration Readiness.*

**H8.**  *Importance of Migration Benefits conveys a positive indirect association between Perceived Institutional and Professional Deficit and Migration Readiness.*

## 3. Materials and Methods

### 3.1. Research Design and Data Collection

The present study aims to investigate the factors associated with the readiness for professional migration among pharmacists working in Romania, regardless of the sector in which they operate. Based on the Push–Pull–Mooring theoretical framework, the proposed conceptual model analyzes the relationships between Work and Pay Push Factors (WPPF), Perceived Institutional and Professional Deficit (PID), Importance of Migration Benefits (IMB), Migration Mooring Barriers (MB) and Migration Readiness (MR).

The questionnaire used in this study was developed by the authors based on the Push–Pull–Mooring theoretical framework and the literature on pharmacist migration, healthcare professional mobility, job satisfaction and pharmaceutical workforce retention. The instrument does not represent a full translation of a previously validated scale, but rather integrates and conceptually adapts dimensions and items identified in previous research, so that they reflect the particularities of the pharmaceutical profession and the Romanian context. The sources used to substantiate each construct and the corresponding indicators are presented in Table 1.

The full questionnaire comprised 62 questions, to which the timestamp automatically generated by the Google Forms platform was added. The instrument included the question on informed consent, socio-demographic and professional characteristics, experience and general orientation regarding migration, motivations for migration, perceived barriers to relocation, current professional conditions, retention measures, general attitudes and a set of items on individual characteristics. Of the 62 questions, 25 items were included in the initial specification of the PPM measurement model. They were distributed as follows: three items for Work and Pay Push Factors (WPPF), five items for Perceived Institutional and Professional Deficit (PID), eight items for Importance of Migration Benefits (IMB), six items for Migration Mooring Barriers (MB) and three items for Migration Readiness (MR). The other questions had a descriptive, exploratory or characterization role for the respondents and were not included in the structural model analyzed by PLS-SEM.

The instrument development process aimed to ensure concordance between the conceptual definition of each construct and the content of the items used to measure it. The wordings were adapted so that they were relevant to the activity of pharmacists in Romania and could be understood independently of the respondents’ familiarity with the literature on professional migration. The items on the benefits of migration were formulated based on research on the pull factors of professional mobility, those on working conditions and remuneration were developed based on the literature on professional satisfaction and retention of pharmacists, and the barriers to migration were developed based on the Push–Pull–Mooring literature and studies on the integration of health professionals into destination systems.

The content validity of the instrument was assessed by a group of seven experts: two university professors in the field of pharmacy, one associate professor, one project manager, two pharmacists with practical experience and one researcher with experience in quantitative research methodology. The evaluators analyzed the relevance of each item to the corresponding construct, the clarity of the formulation, the adequacy of the terminology for the target population and the coverage of the conceptual domain.

Before the main data collection, the preliminary version of the instrument underwent cognitive pretesting with 25 professionally active pharmacists. This stage was conducted as an instrument-development exercise and aimed to assess the clarity and comprehensibility of the items, the appropriateness of the response options, the sequence of questions, and the feasibility of questionnaire administration. Participants were asked to identify wording that they considered ambiguous, difficult to understand, or redundant. Based on their feedback, unclear formulations were revised and additional explanations were introduced for concepts such as international professional mobility, external professional opportunities, and Migration Readiness. The conceptual scope of the constructs and the hypothesized relationships were not modified. The responses obtained during cognitive pretesting were not retained in the analytical dataset, were not combined with the main-study responses, and were not used in the estimation of the measurement or structural models.

Following the two stages of evaluation of the instrument, namely the content validity analysis carried out by experts and the cognitive pretest, the final version of the questionnaire used for data collection was established. The modifications mainly concerned the clarity of the formulations, the adequacy of the terminology and the comprehensibility of the response options and did not modify the conceptual scope of the constructs or the relationships formulated within the theoretical model.

The use of these distribution channels allowed access to pharmaceutical professionals in different occupational and geographical contexts. Similar online recruitment methods have also been used in other research on pharmaceutical services and pharmacist activity. For example, ref. [35] used Facebook, WhatsApp and LinkedIn to collect data on pharmaceutical services in Romania, ref. [36] used these platforms to recruit community pharmacists in Pakistan, and ref. [37] distributed questionnaires via social media in cross-sectional research aimed at pharmacists.

The research used a quantitative, cross-sectional design. The main-study data were collected through an online questionnaire developed in Google Forms between 12 March and 25 May 2026. Recruitment began on the date of the formal ethical approval. The questionnaire was distributed through several digital channels, including e-mail messages sent through pharmacy-graduate and professional networks, professional pharmacist groups on Facebook and WhatsApp, and LinkedIn, as in other studies have been used [38,39,40]. The use of multiple channels was intended to reach professionally active pharmacists working in different occupational and geographical contexts. However, recruitment remained nonprobability-based, and no sampling quotas were applied.

The recruitment strategy was not designed to produce a statistically representative national sample. Participation depended on respondents’ access to the recruitment channels, eligibility, availability, and willingness to complete the questionnaire. Because the invitation was distributed through open and professional-network channels, the number of eligible individuals who viewed the invitation could not be determined. Consequently, a conventional response rate could not be calculated.

The data collected reflect professional evaluations and reported intentions of pharmacists in real work contexts and can be considered observational survey data collected from pharmacists in real-world professional settings. They do not come from an experiment or a controlled intervention, but capture respondents’ perceptions of professional conditions, external opportunities and barriers to relocation in the context of their current practice.

A convenience sampling method was used, with participants recruited based on their availability and accessibility. The target population was represented by professionals active in the pharmaceutical field, as they can directly assess working conditions, remuneration level, institutional support, external professional opportunities and availability to work outside Romania. Before completing the questionnaire, participants were informed about the purpose of the research, the voluntary nature of participation, the anonymity of responses and compliance with personal data protection requirements. The proposed structural model can be seen in Figure 1.

The inclusion criteria were being at least 18 years old, holding a pharmacy qualification, being professionally active in Romania at the time of questionnaire completion, and providing informed consent. Eligibility was assessed using respondents’ self-reported answers to the consent, education, and professional-activity questions. Professional licensing status was not independently verified against an external registry. Accordingly, the analytical population is described as self-identified professionally active pharmacists who reported meeting the study’s eligibility criteria.

The main-study protocol was reviewed and approved by the Research Ethics Committee of the “Grigore T. Popa” University of Medicine and Pharmacy of Iași, Romania (Approval No. 738, dated 12 March 2026). Main-study recruitment and data collection commenced on 12 March 2026 and were completed on 25 May 2026. The study was conducted in accordance with the principles of the Declaration of Helsinki, applicable European and national research-integrity requirements, and the General Data Protection Regulation.

The online form was configured to permit only one submission per respondent. Following data collection, the database was additionally screened manually using the automatically generated timestamps and comparisons of response patterns. No duplicate submissions were identified. A total of 542 complete questionnaires were recorded. All respondents provided informed consent and reported meeting the eligibility criteria. No incomplete questionnaires or responses requiring exclusion were identified; therefore, all 542 questionnaires were retained for analysis.

The sample size was evaluated in relation to the complexity of the structural model and the maximum number of predictors of an endogenous construct. Migration Readiness, the construct with the largest number of predictors, is explained by WPPF, PID, IMB and MB. The sample of 542 respondents provides an adequate basis for estimating structural coefficients, applying the bootstrapping procedure with 10,000 subsamples and predictive evaluation by cross-validation. However, the adequacy of the sample does not eliminate the limitations on representativeness, determined by convenience recruitment.

The analytical sample comprised 542 respondents. Women represented 89.48% of the sample, and 85.42% of respondents resided in urban areas. More than half had over ten years of professional experience, while 33.21% had between four and ten years of experience. Most respondents worked in community pharmacies and had employee status. These characteristics describe the obtained convenience sample and should not be interpreted as estimates of the demographic or professional distribution of the Romanian pharmacist population. The sample therefore primarily reflects the perceptions of experienced, urban, female pharmacists working in community-pharmacy settings.

Sociodemographic and professional variables were collected to characterize the sample but were not included as post hoc control variables in the structural model. The model was specified a priori to test the relationships derived from the Push–Pull–Mooring framework rather than subgroup differences or demographic determinants of migration readiness. No hypotheses concerning direct demographic effects were formulated. In addition, the convenience sample was substantially unbalanced across sex, residence, occupational sector, and professional-status categories, with relatively small numbers in several subgroups. Introducing selected controls after examining the sample and structural results could therefore produce unstable estimates and change the theoretical estimand without a prespecified analytical rationale. Family circumstances were not measured as a separate sociodemographic variable; the questionnaire assessed the perceived importance of family ties and responsibilities as a component of the formative MB composite. The reported structural coefficients should consequently be interpreted as unadjusted associations within the obtained sample.

### 3.2. Data Analysis Method

The analysis was carried out in two main stages. In the first stage, the measurement model was evaluated, by checking the external loadings of the indicators, collinearity, reliability, convergent validity and discriminant validity. For reflective constructs, Cronbach’s Alpha, rho_A, composite reliability rho_C and average variance extracted were analyzed, while for formative constructs, VIF values, external weights, external loadings and their statistical significance were examined. Following the evaluation, only indicators that adequately contributed, both statistically and conceptually, to the measurement of the constructs were retained in the model. Given that the variables analyzed were measured using the same questionnaire and reported by the same respondents, the risk of variation associated with the common measurement method was also taken into account. Several procedural measures were used to reduce this risk. Participation was anonymous and voluntary, respondents were informed that there were no right or wrong answers, and the items were formulated in as clear and neutral a language as possible. The questionnaire was also organized into distinct sections, and the indicators used in the model were measured through several response formats and categories. These measures aimed to reduce the effects generated by social desirability, artificial consistency of responses, and the use of a single collection method. These procedural measures contribute to reducing the risk of common method variance, but do not allow for its complete exclusion. The VIF values reported subsequently were used to assess the collinearity of the measurement and structural models and are not interpreted as a definitive demonstration of the absence of common method bias. The PLS-SEM analysis was conducted in SmartPLS 4 using standardized results and the path-weighting scheme.

Thus, the revised primary model included two indicators for measuring Work and Pay Push Factors (WPPF), five indicators for measuring Perceived Institutional and Professional Deficit (PID), eight indicators for measuring Importance of Migration Benefits (IMB), six indicators for measuring Migration Mooring Barriers (MB), and three indicators for measuring Migration Readiness (MR). PID, IMB, and MR were specified reflectively, whereas WPPF and MB were modeled formatively because their indicators represent distinct dimensions that jointly define the corresponding composites.

The PLS-SEM analysis was conducted in SmartPLS 4 using standardized results and the path-weighting scheme. The algorithm used initial weights of 1.0, a maximum of 3000 iterations, and a stopping criterion of 10^−7^. The primary model comprised five constructs and 24 indicators: two formative WPPF indicators, five reflective PID indicators, eight reflective IMB indicators, six formative MB indicators, and three reflective MR indicators. The structural model included the paths WPPF → IMB, WPPF → MR, PID → IMB, PID → MR, IMB → MR, and MB → MR.

After estimating the measurement and structural models, the internal cross-validated predictive performance of the selected model was assessed using PLSpredict with 10 folds and 10 repetitions [41]. Within each repetition, the fixed PLS-SEM specification was re-estimated using the training folds, and predictions were generated for the corresponding holdout fold. Q^2^ predict, RMSE, and MAE values were examined for the indicators of the two endogenous constructs, and the PLS-SEM prediction errors were compared with those of an indicator-mean benchmark and a linear-model benchmark. CVPAT was additionally used to test differences in predictive loss [42,43].

The study used a quantitative cross-sectional design based on questionnaire data. The proposed measurement and structural models were estimated using PLS-SEM in SmartPLS 4. PLS-SEM was selected because the study examined a model containing both reflective and formative composite specifications and aimed to evaluate theoretically proposed associations and the internal predictive performance of an exploratory model [44,45,46].

Indicator assessment, indicator-retention decisions, and final model specification were completed using the full analytical sample before PLSpredict was applied. No indicator selection was repeated within the training folds. The procedure therefore evaluates the internal cross-validated predictive performance of the selected model, rather than the predictive validity of the complete model-development process. All questionnaires were complete, and no missing-data imputation was performed.

The analytical strategy therefore assessed the measurement model, the hypothesized structural associations, and the internal cross-validated predictive performance of the selected specification. The predictive analysis was not designed to validate the prediction of actual migration behavior or changes in workforce vulnerability over time.

The structural model tested includes the following relationships: WPPF → IMB, WPPF → MR, PID → IMB, PID → MR, IMB → MR and MB → MR. In addition, the indirect effects WPPF → IMB → MR and PID → IMB → MR were analyzed to examine whether Importance of Migration Benefits is involved in the specific indirect associations between the associations of Work and Pay Push Factors and Perceived Institutional and Professional Deficit with Migration Readiness.

### 3.3. Measurement of Constructs

The indicators used in this research were defined according to the nature of the constructs analyzed. Thus, the constructs Perceived Institutional and Professional Deficit (PID), Importance of Migration Benefits (IMB) and Migration Readiness (MR) were operationalized as type A composites, specific to reflective measurement models. In their case, the indicators represent manifestations of latent constructs and are assumed to be correlated and partially interchangeable.

In contrast, Work and Pay Push Factors (WPPF) and Migration Mooring Barriers (MB) were operationalized as type B composites, specific to formative measurement models. In the case of these constructs, the indicators represent distinct dimensions that contribute together to form the composite, without necessarily being interchangeable or strongly correlated [45,47].

The conceptual model included five constructs: work and pay push factors (WPPF), perceived institutional and professional deficit (PID), Importance of Migration Benefits (IMB), Migration Mooring Barriers (MB) and readiness to migrate (MR).

The variables were constructed based on the literature and the theoretical logic of the Push–Pull–Mooring model. According to this framework, readiness to migrate is influenced by the interaction between factors that determine the distance from the current professional situation, the perceived advantages of external opportunities and personal or contextual factors that limit mobility [13,15].

The operationalization of the constructs was also based on research on pharmacist migration, healthcare professional mobility, job satisfaction and retention of the pharmaceutical workforce. Thus, the items on salary, working conditions, external professional opportunities, professional recognition, barriers to relocation and willingness to migrate were adapted or developed based on dimensions identified in previous studies.

The 25 items included in the initial specification of the measurement model used response scales adapted to the content of the questions. Twenty-one items were measured using five-category scales, and four items used four-category ordinal scales. The 25 indicators included in the initial PPM measurement specification were selected before the measurement-model results from the main study were examined. Their selection was based on their conceptual correspondence with the proposed WPPF, PID, IMB, MB, and MR constructs. The expert assessment and cognitive pretest resulted primarily in wording, terminology, and response-option clarifications and did not change the proposed constructs or structural relationships. The subsequent transition from the initial 25-indicator specification to the final 24-indicator specification was conducted using the full analytical sample and is therefore reported transparently as part of the exploratory measurement-model evaluation.

Items IMB1–IMB8, corresponding to questions Q15–Q22, were preceded by the instruction: “Rate on a scale from 1 to 5 how important each reason is to you when making the decision to leave abroad as a pharmacist.” Responses ranged from 1 = “not at all important” to 5 = “very important.” These indicators therefore measure the importance respondents assign to potential migration-related benefits rather than their belief that those benefits are necessarily available abroad.

Items MB1–MB6, corresponding to questions Q24–Q29, used a scale from 1 = “not a difficulty” to 5 = “a very major difficulty.” Indicators PID4, PID5, and MR3, derived from questions Q37–Q39, used five-category response scales. Q30 assessed satisfaction with current working conditions, and Q31 assessed satisfaction with current salary; both indicators were reverse-coded so that higher values represented greater dissatisfaction.

MR1 and MR2 each had four ordered response categories. In the analytical dataset, the categories were coded 1, 2, 4, and 5, with higher values indicating greater proximity to professional migration; no response category was coded as 3. For MR1, the categories ranged from “I do not intend to” to “Yes, I have already taken concrete steps.” For MR2, they ranged from “I do not intend to leave” to “Within the next 12 months.” The common lowest category identifies the absence of migration intention in two related but distinct questions: MR1 concerns consideration and preparation, whereas MR2 concerns the intended timing of departure.

Before estimating the model, the indicators were coded so that higher values reflect a higher level of the corresponding construct. Consequently, indicators relating to satisfaction with working conditions and remuneration, institutional support and valuing the profession were reversely recoded when the initial formulation had a direction opposite to the measured construct.

The instrument was not fully translated from a previous questionnaire. The items were formulated in Romanian by the authors and conceptually adapted based on the specialized literature. WPPF was developed based on [3,4,5,7]; PID was adapted based on [3,4,20,23]; IMB based on [1,3,4]; MB based on [3,4,13,14,30]; and MR based on [3,4,48].

Table 2 presents the constructs used in the research model, the meaning of their indicators, the theoretical sources informing their operationalization, and the measurement properties of the reflective constructs. Following reconsideration of the formative measurement specifications, all six indicators originally proposed for Migration Mooring Barriers were retained. The revised primary model therefore included two indicators for Work and Pay Push Factors, five indicators for Perceived Institutional and Professional Deficit, eight indicators for Importance of Migration Benefits, six indicators for Migration Mooring Barriers, and three indicators for Migration Readiness.

The initial specification of the measurement model included 25 indicators. Following reconsideration of the formative measurement specifications, all six indicators originally proposed for Migration Mooring Barriers were retained in the revised primary model. These indicators capture distinct and non-interchangeable linguistic, regulatory, financial, informational, family, and adaptation-related barriers. Because formative indicators define the content domain of the composite, their retention was guided primarily by content validity rather than exclusively by the statistical significance of their outer weights.

Q23, concerning disappointment with the Romanian healthcare system, was not retained as an indicator of Work and Pay Push Factors. This decision was based on conceptual and measurement considerations. Q23 was administered under a question asking respondents to rate the importance of different reasons when deciding whether to work abroad. It therefore measured the importance attributed to disappointment with the Romanian healthcare system as a migration-related motive rather than the respondent’s current level of dissatisfaction with the healthcare system. By contrast, Q30 and Q31 directly assessed satisfaction with current working conditions and remuneration.

The revised primary model therefore included 24 indicators: two for Work and Pay Push Factors, five for Perceived Institutional and Professional Deficit, eight for Importance of Migration Benefits, six for Migration Mooring Barriers, and three for Migration Readiness. To ensure traceability between the original instrument and the revised model, the original indicator codes were retained. The WPPF construct comprised WPPF2 (Q30) and WPPF3 (Q31), while the MB construct comprised MB1–MB6 (Q24–Q29). The full wording, response scales, coding, and model status of the indicators are presented in Supplementary Table S1.

The WPPF construct was developed based on studies highlighting the role of pay and working conditions in pharmacists’ job dissatisfaction, turnover intention, and migration [3,4,5,7]. It was specified formatively because pay and work conditions dissatisfaction are distinct and non-interchangeable dimensions of job strain. The PID construct was adapted from the literature on institutional support, professional recognition, and development opportunities. Previous studies have highlighted the importance of professional status, skills use, career paths and organizational support for pharmacists’ intention to leave or migrate [3,4,20,23]. The PID was specified reflectively because the indicators were designed as manifestations of a general assessment of perceived institutional and professional deficit. It is assumed that pharmacists who perceive a high level of this deficit tend to simultaneously report insufficient institutional support, low valuation of the profession and limited professional prospects. However, as these dimensions can also capture distinct components of the professional environment, the reflective specification is considered a first operationalization of the construct and requires further validation on independent samples.

The IMB construct captures the importance respondents assign to potential economic, professional, and personal benefits when considering professional migration, including higher salary, better working conditions, professional development, career stability, recognition, family living standards, predictability, and international professional experience [1,3,4]. It does not measure respondents’ certainty that these benefits are objectively available abroad. IMB was specified reflectively because the eight indicators were conceptualized as manifestations of a broader orientation toward the importance of potential migration-related benefits. Respondents for whom such benefits are generally more salient are expected to assign greater importance across several economic, professional, and personal domains. Because the indicators nevertheless cover heterogeneous benefit domains, this reflective specification is considered provisional and requires replication and further measurement evaluation in independent samples.

Migration Readiness was conceptualized as a pre-behavioral orientation toward considering professional migration and potentially progressing toward action, rather than as actual migration behavior. MR1 captures whether respondents have considered working abroad and whether this consideration has progressed toward concrete steps. MR2 captures the temporal proximity of a possible departure. MR3 captures whether migration is regarded as a realistic option for career improvement.

To examine whether the structural results depended on the temporally framed MR2 indicator, the complete model was re-estimated after excluding MR2. The sensitivity model retained MR1 and MR3 as indicators of MR, all six MB indicators, and all structural relationships. The results remained substantively stable. The signs and significance decisions of all direct and indirect associations were unchanged, and R^2^ for MR changed only from 0.261 to 0.266. The complete comparison is reported in Supplementary Table S2.

The three indicators therefore represent related but non-identical aspects of the pre-migration decision process: consideration and preparation, intended timing, and perceived career feasibility. The primary PLS-SEM specification treated these indicators as reflective manifestations of a broader Migration Readiness construct. Because this specification combines different aspects of the migration decision process, it is regarded as a provisional operationalization rather than a definitively validated scale.

Three complementary sensitivity assessments were conducted. First, the structural model was re-estimated after excluding MR2. Second, the response distributions and cross-tabulation of MR1 and MR2 were examined. Third, cumulative-logit models were estimated using MR1 and MR2 separately as ordered outcomes. These analyses assessed whether the substantive conclusions depended on the reflective specification of MR or on treating the response categories as equally spaced.

The initial 25-indicator PPM measurement specification was established before the measurement-model evaluation. However, the final 24-indicator specification resulted from reconsideration of the measurement properties and conceptual alignment of the indicators using the full analytical sample. The measurement models and the structural relationships were therefore evaluated within the same sample, and no independent calibration sample, validation sample, or split-sample construct-validation procedure was used. The expert assessment and cognitive pretest supported content relevance, clarity, and comprehensibility but did not constitute independent statistical validation of the constructs. Consequently, the reported reliability, validity, structural, and predictive results should be interpreted as an exploratory evaluation of the selected specification rather than as definitive validation of the instrument. PLSpredict and CVPAT assess the internal cross-validated predictive performance of this selected model but do not provide independent validation of the measurement-development process. Because the full analytical sample had already informed indicator assessment and final model specification, a retrospective random division of the current dataset would not produce a genuinely independent validation sample. An adequately designed calibration/validation procedure would require all item-assessment and model-refinement decisions to be restricted to a calibration sample, followed by evaluation of the unchanged specification in a previously untouched validation sample or in a newly recruited independent sample.

## 4. Results and Discussion

### 4.1. Evaluation of the Measurement Model

In the first stage, the measurement model was evaluated by analyzing external loadings, internal consistency, convergent and discriminant validity, as well as collinearity. This stage is necessary before interpreting the structural model, as it allows checking the quality of the indicators and constructs included in the PLS-SEM analysis [45,46,49].

Since the model includes reflective and formative constructs, distinct criteria were applied. For the reflective constructs PID, IMB and MR, external loadings, Cronbach’s Alpha, rho_A, composite reliability rho_C and AVE were evaluated. For the formative constructs WPPF and MB, collinearity, weights and external loadings, as well as their statistical significance, were analyzed.

#### 4.1.1. Evaluation of Reflective Constructs

The reflective measurement models displayed positive and statistically significant outer loadings. The loadings ranged from 0.713 to 0.795 for PID, from 0.623 to 0.837 for IMB, and from 0.813 to 0.880 for MR. All reflective indicator loadings were statistically significant at *p* < 0.001. Complete indicator-level results, including bootstrap *t*-values, *p*-values, and percentile confidence intervals, are reported in Supplementary Table S3.

IMB8 had an outer loading of 0.623, below the commonly recommended value of 0.708. It was retained because its loading was positive and statistically significant, its removal would not materially improve composite reliability or AVE, and it captures the importance of international professional collaborations and experiences, a conceptually relevant component of IMB.

Internal consistency and convergent validity were satisfactory. Cronbach’s alpha was 0.814 for PID, 0.909 for IMB, and 0.807 for MR. The rho_A values were 0.827, 0.915, and 0.810, respectively, while rho_C was 0.869 for PID, 0.927 for IMB, and 0.886 for MR. AVE was 0.571 for PID, 0.614 for IMB, and 0.721 for MR. All rho_A, rho_C, and Cronbach’s alpha values exceeded 0.70, and all AVE values exceeded 0.50, confirming convergent validity [44,45].

#### 4.1.2. Evaluation of Formative Constructs

For WPPF and MB, Cronbach’s Alpha, composite reliability or AVE were not calculated because the indicators represent distinct dimensions that form the constructs and not interchangeable manifestations of them, as presented in Table 3.

The outer VIF values were 1.127 for the WPPF indicators and ranged from 1.173 to 2.181 for the MB indicators. All values were below 3, providing no indication of critical collinearity among the formative indicators [45,46]. These VIF values assess collinearity within the formative measurement specifications. The threshold of 3.3 discussed in Reference [50] was proposed in the context of the full-collinearity approach to common method bias and was not treated as an equivalent criterion for evaluating collinearity among formative indicators. Accordingly, the reported outer VIF values are not interpreted as demonstrating the absence of common method bias.

For WPPF, both indicators had positive and statistically significant outer weights. Dissatisfaction with working conditions had an outer weight of 0.354 (*p* < 0.001) and an outer loading of 0.631 (*p* < 0.001), while dissatisfaction with remuneration had an outer weight of 0.824 (*p* < 0.001) and an outer loading of 0.943 (*p* < 0.001). These findings indicate that both indicators contributed to the formative composite, with remuneration dissatisfaction displaying the larger relative contribution. The weights represent relative contributions within the composite and should not be interpreted as causal effects or as evidence that one intervention would necessarily be more effective than another.

For Migration Mooring Barriers, family ties and responsibilities had the largest positive outer weight (0.704, *p* < 0.001), followed by uncertainty regarding adaptation (0.442, *p* = 0.004) and relocation and adaptation costs (0.343, *p* = 0.046). The language barrier had a nonsignificant outer weight (0.097, *p* = 0.500) but a positive and statistically significant outer loading (0.321, *p* = 0.010), indicating an absolute contribution to the composite.

Qualification-recognition procedures displayed a significant negative relative weight (−0.336, *p* = 0.039) but a small and nonsignificant outer loading (0.074, *p* = 0.573). Difficulty obtaining information about the employment process also displayed a negative but nonsignificant relative weight (−0.219, *p* = 0.210), while its outer loading was positive and marginally significant (0.247, *p* = 0.049). These negative relative weights should not be interpreted as indicating that regulatory or informational barriers facilitate migration. In a formative composite, outer weights represent the relative contribution of each indicator after accounting for the remaining indicators and may be influenced by shared variance among related barriers.

All six indicators were retained because they represent conceptually distinct and non-interchangeable linguistic, regulatory, financial, informational, family, and adaptation-related barriers. Their retention preserves the intended content domain of the formative composite. Individual outer weights are therefore not interpreted as causal effects or as evidence that one barrier represents a more effective target for intervention than another.

The convergent validity of the formative constructs was not assessed through redundancy analysis because the questionnaire did not include separate global indicators for WPPF and MB. Consequently, the measurement evaluation of these formative composites remains incomplete, and their specification should be regarded as provisional until it is confirmed using redundant global measures and independent samples.

#### 4.1.3. Evaluation of Collinearity of Indicators

Continuing the analysis of the outer model, the VIF values of all indicators are between 1.127 and 3.002. The highest value was recorded for IMB4, namely 3.002, but this was lower than the conservative threshold of 3.3. Therefore, no collinearity problems were identified at the level of the outer model (Table 4).

#### 4.1.4. Assessment of Discriminant Validity

The discriminant validity of reflective constructs was assessed using the Fornell–Larcker criterion and the Heterotrait–Monotrait (HTMT) ratio. The formative constructs WPPF and MB were not included in this assessment, as the Fornell–Larcker and HTMT criteria are developed for reflective measurement models.

According to the Fornell–Larcker criterion, the square root of the AVE value for each construct, presented on the diagonal of the matrix, is greater than its correlations with the other constructs. Thus, the value on the diagonal is 0.849 for MR, 0.784 for IMB, and 0.756 for PID, all of which are superior to the correlations between the constructs. The results indicate that each reflective construct captures a distinct empirical concept.

The HTMT values range from 0.253 to 0.371 and are considerably lower than the recommended threshold of 0.85. The highest HTMT value was identified between IMB and MR, at 0.371. This association is conceptually logical, since the favorable assessment of the benefits of migration is related to the respondents’ willingness to analyze an external professional opportunity. However, the low value shows that the two constructs remain empirically distinct. The use of the HTMT criterion is recommended in the literature as a robust method for assessing discriminant validity in reflective measurement models [51]. Overall, the reflective constructs show adequate levels of internal consistency, convergent validity, and discriminant validity. For the formative constructs, the results support the absence of problematic collinearity and the statistical or conceptual relevance of the retained indicators. However, the convergent validity of the WPPF and MB could not be assessed by redundancy analysis because the instrument did not include redundant global indicators. Consequently, the validation of the formative measurement model must be considered incomplete and requires confirmation in future research (Table 5). Accordingly, the measurement results reported in this section should be interpreted as within-sample diagnostics of the provisional specification. They do not constitute independent confirmation of construct validity.

### 4.2. Structural Model Evaluation and Hypothesis Discussion

In the second stage of the analysis, the structural model was evaluated by examining the collinearity between the predictor constructs, the path coefficients, the *t*-values, the *p*-values, the confidence intervals, the coefficients of determination R^2^, the effect sizes f^2^, and the specific indirect effects. The significance of the relationships between the constructs was tested by the bootstrapping procedure, using 10,000 subsamples, percentile 95% confidence intervals, a two-tailed significance test, and a fixed random seed. This procedure is frequently used in PLS-SEM analyses to verify the statistical significance of the direct and indirect relationships between the constructs [44,46].

Because the underlying constructs have not been validated in an independent sample, the structural coefficients reported below are conditional on the selected provisional measurement specification. They are therefore interpreted as exploratory, hypothesis-generating associations rather than as confirmatory estimates based on independently validated constructs.

Before interpreting the path coefficients, collinearity among the predictor constructs was examined. The inner VIF values ranged from 1.024 to 1.537, remaining below the conservative threshold of 3.3. Therefore, no problematic collinearity was identified among the predictors of IMB and MR. The model explained 22.8% of the variance in Importance of Migration Benefits (R^2^ = 0.228) and 26.1% of the variance in Migration Readiness (R^2^ = 0.261). The adjusted R^2^ values were 0.225 for IMB and 0.256 for MR.

The explanatory power of the model can be considered modest, but relevant for a complex behavioral phenomenon. Migration Readiness is not solely associated with working conditions, remuneration, or external opportunities, but may also be related to individual characteristics, family situation, professional experiences, intended destination and the concrete possibility of relocation. The literature on health professional migration also shows that mobility decisions result from a combination of economic, professional, personal and institutional factors [1,3,4].

Values displayed on the structural paths (Figure 2) are standardized path coefficients. R^2^ values are reported within the endogenous constructs. Solid lines indicate statistically significant associations at *p* < 0.05, while dashed lines indicate nonsignificant associations. Indicator-level loadings and formative weights are reported separately in Table 2 and Table 3, and Supplementary Table S3.

The SRMR value for the estimated model was 0.056, indicating an acceptable approximate model fit. However, SRMR is an in-sample approximate fit measure and should not be interpreted as evidence of out-of-sample predictive performance, which was assessed separately using PLSpredict and CVPAT.

#### 4.2.1. Association Between Work and Pay Push Factors and Importance of Migration Benefits

The first hypothesis examined the association between Work and Pay Push Factors and Importance of Migration Benefits. The results indicated a positive and statistically significant association (β = 0.469; t = 7.542; *p* < 0.001; 95% CI [0.339, 0.584]) (see Table 6), supporting H1. Respondents who reported greater dissatisfaction with their current working conditions and remuneration also assigned greater importance to the potential professional and economic benefits of working abroad. The effect size was moderate (f^2^ = 0.228) and represented the largest positive effect size identified in the structural model.

This pattern is consistent with previous research linking dissatisfaction with salary and working conditions to the evaluation of external professional opportunities [3,4,5,7]. Within the PPM framework, the result is compatible with an association between unfavorable evaluations of the current professional situation and the importance assigned to potential migration benefits. However, because the data are cross-sectional, the result does not demonstrate that dissatisfaction produces a subsequent change in respondents’ evaluation of external opportunities.

#### 4.2.2. Association Between Work and Pay Push Factors and Migration Readiness

The second hypothesis examined the direct association between Work and Pay Push Factors and Migration Readiness. The results indicated a positive and statistically significant association (β = 0.189; t = 3.924; *p* < 0.001; 95% CI [0.092, 0.280]), supporting H2. The effect size was small (f^2^ = 0.032). Thus, greater dissatisfaction with current working conditions and remuneration was associated with higher Migration Readiness.

The positive association between WPPF and MR is consistent with studies showing that insufficient remuneration, high workload, poor work–life balance and limited development opportunities favour the intention to emigrate of pharmacists and other health professionals [6,27]. In Romania, ref. [10] highlights the increased dissatisfaction of pharmacy graduates with the equity of remuneration and professional prospects, confirming the existence of pressures that can reduce attachment to the profession and encourage the evaluation of external opportunities.

However, the small effect size, f^2^ = 0.032, shows that dissatisfaction with work and pay is associated with Migration Readiness, but does not explain it independently. In the logic of the PPM, push factors are associated with the consideration of migration, but the association between dissatisfaction and Migration Readiness also depends on the perceived benefits of working abroad and on personal or contextual barriers to relocation.

#### 4.2.3. Association Between Perceived Institutional and Professional Deficit and Importance of Migration Benefits

The third hypothesis analyzed the relationship between perceived institutional and professional deficit and Importance of Migration Benefits. The results indicate a very small and statistically insignificant positive relationship: β = 0.019; t = 0.366; *p* = 0.714. The confidence interval includes the zero value, and the effect size is negligible, f^2^ < 0.001. Therefore, H3 is not supported. The result indicates that perceptions of low institutional support, insufficient professional recognition, and limited development prospects are not independently associated with Importance of Migration Benefits in the estimated model.

The lack of support for the PID → IMB relationship is compatible with studies showing that institutional support, professional recognition and career opportunities do not always directly generate the intention to leave or the favorable evaluation of external alternatives. These dimensions may instead influence satisfaction, organizational involvement, professional attachment, and retention. Previous studies [52,53,54] have reported limited or indirect associations between institutional and professional factors and migration-related intentions, while ref. [55] highlighted the role of institutional support in facilitating integration and retention.

A possible explanation is that PID captures general assessments of professional recognition, institutional representation, and development prospects, whereas IMB captures the importance assigned to potential economic, professional, and personal benefits when considering migration. A pharmacist may therefore report dissatisfaction with institutional or professional conditions without necessarily assigning greater importance to migration-related benefits. The nonsignificant PID → IMB association does not imply that institutional conditions are unimportant for pharmacists’ satisfaction or retention. Moreover, the statistical significance of the WPPF → IMB association and the nonsignificance of the PID → IMB association do not, by themselves, demonstrate that the two coefficients differ significantly, as no formal comparison between them was conducted.

#### 4.2.4. Association Between Perceived Institutional and Professional Deficit and Migration Readiness

The fourth hypothesis analyzed the relationship between perceived institutional and professional deficit and readiness to migrate. Although the coefficient is positive, the effect is not statistically significant at the 5% level (β = 0.073; t = 1.730; *p* = 0.084), the confidence interval includes the zero value, and f^2^ = 0.006 indicates a negligible effect. Therefore, H4 is not supported.

The result shows that perceptions of low institutional support, insufficient professional recognition, and limited development prospects are not directly associated with pharmacists’ Migration Readiness in the estimated model. This finding is consistent with studies showing that institutional factors influence satisfaction, organizational commitment, or intention to leave through intermediary mechanisms rather than a direct effect on mobility [26,56].

A possible explanation is that PID reflects general evaluations of the professional system, while MR implies a more concrete orientation towards migration. This also depends on the existence of external opportunities, language skills, recognition of qualifications, financial resources, and family responsibilities [57]. Thus, a pharmacist may perceive institutional deficiencies without being prepared to analyze or initiate migration.

The lack of support for H4 does not diminish the relevance of PID for job satisfaction or retention, but indicates that, in the analyzed model, it does not have a sufficient direct and independent contribution to explaining the readiness for migration. Associations between institutional conditions and migration-related outcomes may involve other variables not included in the present model, such as job satisfaction, organizational commitment, or professional burnout.

#### 4.2.5. Association Between Importance of Migration Benefits and Migration Readiness

The fifth hypothesis examined the relationship between Importance of Migration Benefits and Migration Readiness. The results indicated a positive and statistically significant association (β = 0.223; t = 5.539; *p* < 0.001; 95% CI [0.141, 0.300]), supporting H5. The effect size was small (f^2^ = 0.052). Respondents who assigned greater importance to economic, professional, career, family, and organizational benefits when considering work abroad also reported higher Migration Readiness.

The result is consistent with the role of pull factors and is consistent with studies showing that educational opportunities, better working and living conditions, salaries and career advancement prospects favor the migration intention of health professionals [31,32]. At the same time, mobility is influenced by the feasibility of relocation. Family ties and personal obligations can reduce migration intention [31,32], and language skills, the existence of relatives abroad and previous international experience can facilitate the transformation of interest in migration into a concrete option [57].

Therefore, although external benefits are positively associated with Migration Readiness, this association also depends on the intervening obstacles and anchoring factors described by [13,14]. Pharmacists may evaluate external opportunities favorably without having the resources, family support or availability necessary to initiate the migratory process.

#### 4.2.6. Association Between Migration Mooring Barriers and Migration Readiness

The sixth hypothesis examined the relationship between Migration Mooring Barriers and Migration Readiness. The results indicated a negative and statistically significant association (β = −0.326; t = 9.024; *p* < 0.001; 95% CI [−0.406, −0.264]), supporting H6. The effect size was small to moderate (f^2^ = 0.140). Therefore, higher levels of the formative barrier composite were associated with lower Migration Readiness. This coefficient represents the association between the overall MB composite and MR and does not demonstrate separate structural effects for each individual barrier.

Within the formative MB composite, family responsibilities and uncertainty regarding adaptation displayed the largest positive relative weights. These weights describe the relative contribution of each indicator to the composite under the estimated specification; they should not be interpreted as separate causal effects, as evidence that the corresponding barriers are more important in an absolute sense, or as a ranking of policy interventions. The negative MB → MR coefficient indicates that higher levels of the overall linguistic, regulatory, financial, informational, family, and adaptation-related barrier composite were associated with lower Migration Readiness. This finding is consistent with research highlighting the relevance of language, cultural integration, social relationships, family circumstances, and professional support for the international mobility of healthcare professionals [30].

#### 4.2.7. The Indirect Association Through Importance of Migration Benefits

The specific indirect association WPPF → IMB → MR was positive and statistically significant (β = 0.105; t = 4.609; *p* < 0.001; 95% CI [0.062, 0.151]), supporting H7. The direct WPPF → MR association also remained positive and statistically significant, while the total association between WPPF and MR was β = 0.294 (t = 6.678; *p* < 0.001). This pattern is statistically compatible with complementary partial mediation. However, given the cross-sectional design, it should be interpreted as a direct association accompanied by a statistically significant specific indirect association, rather than as evidence that dissatisfaction changes migration-related benefit evaluations and subsequently produces Migration Readiness over time.

By contrast, the specific indirect association PID → IMB → MR was not statistically significant (β = 0.004; t = 0.363; *p* = 0.716; 95% CI [−0.016, 0.031]); therefore, H8 was not supported. The total association between PID and MR was also nonsignificant (β = 0.077; t = 1.813; *p* = 0.070). These findings do not indicate that institutional and professional conditions are irrelevant to pharmacists’ professional experiences or retention. They show only that the present model did not identify statistically significant direct or IMB-related indirect associations between PID and Migration Readiness. Associations with migration-related outcomes may involve other variables, such as job satisfaction, organizational commitment, burnout, perceived equity, or intention to leave the current position.

### 4.3. Robustness Assessment of the Migration Readiness Specification

Because the primary Migration Readiness specification combines consideration of migration, intended timing, and perceived career feasibility, three complementary analyses were conducted. The PLS-SEM model was re-estimated without MR2, the response distributions and cross-tabulation of MR1 and MR2 were examined, and cumulative-logit models were estimated using MR1 and MR2 separately as ordered outcomes.

After excluding MR2, the principal structural conclusions remained stable. WPPF remained positively associated with MR (β = 0.177; t = 3.326; *p* < 0.001; 95% CI [0.070, 0.277]), IMB remained positively associated with MR (β = 0.263; t = 6.210; *p* < 0.001; 95% CI [0.177, 0.342]), and MB remained negatively associated with MR (β = −0.317; t = 8.880; *p* < 0.001; 95% CI [−0.395, −0.256]). The PID → MR association remained nonsignificant (β = 0.053; t = 1.231; *p* = 0.218; 95% CI [−0.029, 0.142]). The specific indirect association WPPF → IMB → MR also remained statistically significant (β = 0.123; t = 4.918; *p* < 0.001; 95% CI [0.076, 0.174]), whereas the PID → IMB → MR indirect association remained nonsignificant (β = 0.005; t = 0.357; *p* = 0.721; 95% CI [−0.019, 0.036]). The explained variance in MR changed only slightly, from R^2^ = 0.261 in the primary model to R^2^ = 0.266 in the model without MR2, while the estimated-model SRMR changed from 0.056 to 0.055. These results indicate that the main structural conclusions are not dependent on the inclusion of MR2.

For MR1, 183 respondents (33.76%) selected the no-intention category, 92 (16.97%) were unsure, 218 (40.22%) considered migration as a future possibility, and 49 (9.04%) reported having already taken concrete steps. For MR2, 271 respondents (50.00%) selected no intended departure, 97 (17.90%) selected departure after 4–5 years, 120 (22.14%) selected departure within the next 2–3 years, and 54 (9.96%) selected departure within the next 12 months.

The two indicators were strongly associated (Spearman’s ρ = 0.788, *p* < 0.001). Among the 183 respondents who selected no migration intention on MR1, 182 (99.5%) also selected no intended departure on MR2. Among the 49 respondents who reported having taken concrete steps on MR1, 36 (73.5%) indicated an intended departure within the next 12 months on MR2. The intermediate categories did not overlap completely, supporting the interpretation that MR1 and MR2 capture related but non-identical aspects of Migration Readiness, all the details can be seen in Table 7.

The complementary ordinal logistic regression models produced the same substantive pattern for both ordered outcomes. Higher WPPF and IMB scores were associated with higher MR1 and MR2 response categories, whereas MB was negatively associated with both ordered outcomes. PID was not statistically significant at the 5% level in either model. These results indicate that the main substantive conclusions do not depend solely on treating the four response categories as equally spaced, all the details can be seen in Table 8.

### 4.4. Internal Cross-Validated Predictive Assessment

In addition to evaluating the structural associations, the internal cross-validated predictive performance of the selected model was assessed using PLSpredict. The analysis used 10 folds and 10 repetitions and focused on the indicators of the two endogenous constructs, IMB and MR. Because indicator assessment and model specification were completed using the full sample before cross-validation, the results evaluate the fixed selected model rather than the complete procedure through which the model was developed.

The results indicated positive Q^2^ predict values for all indicators of the endogenous constructs, ranging from 0.073 to 0.171. At the construct level, Q^2^ predict was 0.210 for IMB and 0.193 for MR. These positive values indicate that the PLS-SEM predictions produced lower prediction errors than the naive indicator-mean benchmark, supporting the model’s out-of-sample predictive relevance within the cross-validation procedure.

The PLS-SEM model produced lower RMSE values than the linear model for all 11 indicators, although the differences were small for several indicators. For MAE, PLS-SEM produced lower values for nine of the 11 indicators. IMB7 and IMB8 were the exceptions, for which the linear model produced marginally lower MAE values. Overall, the results indicate modest but consistent predictive performance relative to the linear benchmark, as can be seen in Table 9.

The predictive evaluation was completed by CVPAT. This test compares the predictive loss of the PLS-SEM model with that of a benchmark based on the average of the indicators and with that of a reference linear model. A negative and significant difference indicates a superior predictive performance of the PLS-SEM model.

The CVPAT results are presented in Table 10. At the construct level, the PLS-SEM predictive loss was significantly lower than the indicator-mean benchmark for both IMB (ΔLoss = −0.100; t = 2.811; *p* = 0.005) and MR (ΔLoss = −0.279; t = 5.386; *p* < 0.001). The PLS-SEM predictive loss was also significantly lower than that of the linear model for IMB (ΔLoss = −0.020; t = 2.611; *p* = 0.009) and MR (ΔLoss = −0.035; t = 3.151; *p* = 0.002). At the overall-model level, the mean loss difference was significant relative to both the indicator-mean benchmark (ΔLoss = −0.149; t = 4.705; *p* < 0.001) and the linear model (ΔLoss = −0.024; t = 3.767; *p* < 0.001). These results support out-of-sample predictive performance within the applied cross-validation procedure, although the magnitude of the predictive advantage over the linear model was modest.

The PLSpredict and CVPAT results support the internal cross-validated predictive performance of the selected model for questionnaire responses within the analyzed dataset. Positive Q^2^ predict values were obtained for all endogenous indicators, and PLS-SEM produced lower RMSE values than the linear benchmark for all 11 indicators. However, the differences from the linear model were small for several indicators, indicating only a modest incremental predictive advantage.

These results should not be extended to the prediction of actual migration behavior or workforce changes. The holdout observations were drawn from the same convenience sample, and the outcome was Migration Readiness rather than subsequent migration. Moreover, indicator assessment and model specification preceded cross-validation and were not repeated within the training folds. The analysis therefore validates neither the complete model-development process nor the use of the instrument for longitudinal workforce monitoring.

## 5. Conclusions

This research examined the factors associated with pharmacists’ Migration Readiness in Romania by integrating push, pull, and mooring factors. Within the analyzed sample, the model accounted for 22.8% of the variance in Importance of Migration Benefits and 26.1% of the variance in Migration Readiness. Because the constructs have not been independently validated, these estimates and the associated structural relationships are conditional on the selected provisional measurement specification and should be regarded as hypothesis-generating.

Beyond its explanatory results, the selected model displayed internal cross-validated predictive performance for held-out questionnaire responses. Q^2^ predict values were positive for all indicators of the endogenous constructs, and the model produced lower RMSE values than the linear benchmark. CVPAT also indicated statistically lower predictive loss than both benchmarks. Nevertheless, the incremental advantage over the linear model was modest, and the findings apply only to questionnaire responses from folds drawn from the same convenience sample. They do not demonstrate temporal prediction, external transportability, or prediction of actual migration.

The strongest direct association with Migration Readiness was observed for the overall Migration Mooring Barriers composite, with a negative coefficient. This finding indicates that the combined linguistic, regulatory, financial, informational, family, and adaptation-related constraints were associated with lower readiness to migrate. However, the formative outer weights represent relative contributions within the composite and should not be interpreted as separate causal effects or as a ranking of potential policy interventions. The results therefore support the relevance of relocation feasibility as a broader domain but do not establish which specific intervention would most effectively influence migration readiness.

Dissatisfaction with working conditions and remuneration was positively associated with both Importance of Migration Benefits and Migration Readiness. The WPPF → IMB relationship represented the largest positive structural coefficient in the model. Respondents reporting greater dissatisfaction with their current working conditions and remuneration also assigned greater importance to potential migration-related benefits. This association does not demonstrate that dissatisfaction causes a change in the evaluation of external opportunities.

Importance of Migration Benefits was positively associated with Migration Readiness. Respondents who assigned greater importance to potential economic, professional, career, family, and organizational benefits when considering migration also reported higher Migration Readiness. IMB measures the importance assigned to these potential benefits; it does not establish that respondents believed that the benefits were objectively available in a particular destination country. The small effect size also indicates that IMB alone provides only a limited explanation of individual differences in Migration Readiness.

A positive and statistically significant specific indirect association was identified for WPPF → IMB → MR. The direct WPPF → MR association also remained statistically significant. This pattern is compatible with complementary partial mediation in a statistical sense, but the cross-sectional data do not establish a temporal sequence or a causal mechanism linking dissatisfaction, benefit evaluation, and Migration Readiness.

The associations of PID with IMB and MR were not statistically significant, and the specific indirect association through IMB was also nonsignificant. These findings do not demonstrate that institutional support, professional recognition, or development opportunities are unimportant for pharmacists’ satisfaction, retention, or other professional outcomes. They indicate only that PID did not display statistically significant independent associations with IMB or MR in the estimated model. Furthermore, the results do not establish that the PID coefficients differ significantly from the corresponding WPPF coefficients, because no formal coefficient-comparison test was conducted. Overall, the findings should be interpreted as cross-sectional statistical associations rather than as evidence of causal relationships or subsequent migration behavior.

### 5.1. Theoretical Implications

The main theoretical contribution is the application of the Push–Pull–Mooring model in the analysis of the readiness for migration of pharmacists in Romania, a context insufficiently investigated through an integrated model. The research brings together in a single framework the professional conditions in the country of origin, the benefits attributed to external destinations and the personal barriers to relocation.

The results support the interdependence between push and pull factors. Dissatisfaction with work and remuneration is not only directly associated with the readiness for migration, but also with the evaluation of external benefits. Testing the specific indirect association through IMB shows that WPPF is associated with MR both directly and through a statistical pathway involving the importance assigned to migration benefits; it does not establish a temporal or causal mechanism.

The results also support the distinct role of mooring factors. Linguistic, regulatory, informational, financial, family, and adaptation-related barriers represent a domain that is conceptually separate from the importance assigned to migration benefits. The negative MB → MR coefficient indicates that the overall feasibility of relocation is associated with Migration Readiness. However, the cross-sectional design and formative specification do not permit causal conclusions regarding the effects of individual barriers.

The insignificance of the PID relationships delimits the professional factors immediately felt by general institutional assessments. The results do not support the automatic treatment of the perception of institutional deficit as a direct predictor of international mobility. Associations between institutional conditions and migration-related outcomes may involve variables not included in the present model, such as job satisfaction, professional commitment, or burnout.

An additional contribution consists of delimiting the readiness for migration from the actual migratory behavior. MR captures the openness, evaluation and predisposition to initiate mobility, without implying the subsequent occurrence of migration. At the same time, the use of reflective and formative constructs allows for the differentiated representation of the variables: WPPF and MB are made up of distinct dimensions, while PID, IMB and MR are measured by manifestations of the corresponding constructs.

### 5.2. Practical Implications

The practical implications should be interpreted within the limits of the cross-sectional nature of the research and the small to moderate effect sizes. The results do not allow the conclusion that a particular intervention will prevent migration, but indicate the areas most clearly associated with readiness for mobility.

First, the results identify working conditions and remuneration as relevant domains for further investigation in pharmacist-retention research. Depending on the issues identified, adjustment of responsibilities, schedule predictability, shift flexibility, transparent salary criteria and benefits linked to experience and performance can be analyzed.

Second, the WPPF → IMB and IMB → MR associations indicate that retention does not depend exclusively on salary level. Development opportunities, stability and professional recognition are part of the benefits attributed to working abroad. Employers can therefore strengthen career paths, access to training, mentoring and the use of pharmacists’ skills. These measures are justified by the dimensions of IMB included in the research, without assuming that each intervention will directly reduce migration.

Third, the MB → MR association indicates that retention policies should consider the broader feasibility of relocation, including family responsibilities, adaptation uncertainty, language proficiency, regulatory procedures, access to employment information, and relocation costs. The present results do not demonstrate that intervening in any single barrier would reduce migration readiness, nor do the relative formative weights identify the most effective policy intervention. These dimensions should instead be regarded as areas for further investigation and for the development of context-sensitive support measures.

The dimensions examined in this study may inform the design of future anonymous organizational surveys concerning working conditions, migration-related considerations, and relocation barriers. However, the present instrument should not yet be used to classify individual employees, estimate individual migration risk, or guide personnel decisions. Any future organizational application would require independent validation and strict safeguards regarding confidentiality and nonpunitive use.

From an applied perspective, the model provides a candidate framework for future research on pharmaceutical-workforce retention. Its constructs may be examined in repeated and representative surveys to determine whether they are stable over time and whether changes in their scores correspond to subsequent workforce outcomes. The present cross-sectional findings alone do not validate the model as a monitoring or decision-support instrument.

From a health-system management perspective, the findings identify domains that merit further investigation, including dissatisfaction with working conditions and remuneration, the importance assigned to migration benefits, relocation barriers, and Migration Readiness. Before these dimensions can be incorporated into workforce-monitoring activities, further studies must establish their temporal stability, measurement invariance, external validity, and association with subsequent migration behavior. The present results should therefore be used to formulate future research questions rather than to prioritize or evaluate current workforce interventions.

### 5.3. Research Limitations and Future Directions

This study has several limitations that should be considered when interpreting the findings. First, it used a nonprobability convenience sample recruited through online channels and professional networks. The number of eligible pharmacists who received or viewed the invitation could not be established, and a conventional response rate could not be calculated. Individuals with a stronger interest in professional migration may have been more likely to participate, creating a risk of self-selection and nonresponse bias. The sample was also concentrated among women, urban residents, experienced professionals, employees, and community pharmacists. Consequently, the sample cannot be considered statistically representative of all pharmacists in Romania, and neither the descriptive distributions nor the structural coefficients should be generalized to the national pharmacist population.

Second, the sample structure is characterized by a high share of female respondents, people from urban areas, and pharmacists working in community pharmacies. Although this distribution partially reflects the characteristics of the pharmaceutical sector, the results may capture to a greater extent the perceptions of these professional categories. Future research should use probability or stratified samples and ensure a more balanced representation of different sectors of activity, geographical regions and occupational categories.

Third, the cross-sectional design of the research does not allow for the establishment of a temporal order between the analyzed constructs nor the formulation of causal conclusions. The direct and indirect relationships identified should be interpreted as statistical associations compatible with the proposed theoretical model. In particular, the indirect effect WPPF → IMB → MR does not demonstrate that job dissatisfaction produces a change in perceived benefits and subsequently in migratory behavior over time. Longitudinal studies could more adequately test these relationships and analyze the evolution of readiness to migrate over time.

Fourth, the information was obtained through self-report and through a single data source. Although procedural measures were used in the construction and administration of the questionnaire to reduce the risk associated with the common measurement method, the influence of social desirability, subjective perceptions or other sources of measurement error cannot be completely ruled out. In the absence of a marker variable or a design with different sources and collection times, the influence of the common method cannot be definitively excluded.

Fifth, Migration Readiness measures respondents’ willingness to consider and initiate migration, not actual migration behavior. Intention or readiness for mobility does not necessarily lead to relocation, as the final decision may depend on concrete employment opportunities, family situation, financial resources, recognition of qualifications and changing professional circumstances. Future studies could follow respondents longitudinally to determine to what extent declared readiness subsequently translates into actual migration.

Sixth, the measurement instrument was developed or conceptually adapted for the specific research context rather than adopted in full from previously validated scales. Expert assessment and cognitive pretesting supported the content relevance, clarity, and comprehensibility of the items, but these procedures do not establish construct validity in an independent sample. Moreover, the final measurement specification was refined and evaluated using the same convenience sample that was subsequently used to estimate the structural relationships. The reported internal-consistency, convergent-validity, discriminant-validity, and structural-model results may therefore reflect sample-specific characteristics. The convergent validity of the formative WPPF and MB composites also remains incomplete because the questionnaire did not include redundant global measures. All structural and predictive findings are conditional on these provisional measurement specifications. Independent-sample replication, confirmatory evaluation of the reflective specifications, redundancy analysis for the formative composites, and measurement-invariance testing are required before the instrument can be considered validated.

The structural model did not include sociodemographic or professional control variables. These characteristics were collected for sample description, while the structural model was specified to test the hypothesized PPM relationships. Because no demographic effects were prespecified and the convenience sample was markedly unbalanced across several categories, post hoc adjustment was not introduced. Therefore, the reported coefficients represent unadjusted associations and may partly reflect the composition of the obtained sample. Future studies should prespecify theoretically justified covariates, recruit sufficiently large and balanced subgroups, and examine measurement invariance and structural differences across age, experience, occupational sector, professional status, and family circumstances.

The convergent validity of the formative WPPF and MB composites could not be assessed through redundancy analysis because the questionnaire did not include separate global indicators representing the same constructs. The formative indicators were evaluated using collinearity statistics, outer weights, outer loadings, bootstrap inference, and content-validity considerations. Although retaining all six MB indicators preserves the intended breadth of the barriers composite, the negative relative weights observed for the regulatory and informational indicators require cautious interpretation. The formative measurement models should therefore be regarded as provisionally evaluated and require further assessment using redundant global measures, independent samples, and longitudinal designs.

Future validation research should use either a newly recruited independent sample or a prospectively planned calibration/validation design. In the latter case, item assessment and measurement-model refinement should be conducted exclusively in the calibration sample, while the resulting fixed specification should be evaluated without further modification in a previously untouched validation sample. The questionnaire should also include theoretically aligned global measures for WPPF and MB so that the convergent validity of these formative composites can be assessed through redundancy analysis. Confirmatory evaluation of the reflective specifications and measurement-invariance testing should precede further interpretation or comparison of the structural relationships.

Furthermore, the explanatory power of the model for readiness for migration is moderate, indicating the existence of additional factors that were not included in the present research. Future models could integrate variables such as job satisfaction, burnout, organizational commitment, perceived equity, family characteristics, prior international experience, the existence of social networks abroad, and specific characteristics of the intended destination.

Finally, PLSpredict and CVPAT provided only an internal cross-validated assessment of the selected model. Indicator assessment and final specification were completed using the full sample before cross-validation; consequently, the analysis does not validate the complete model-development procedure. In addition, the holdout observations originated from the same convenience sample, and the predicted outcomes were questionnaire responses rather than subsequent migration events. External validation requires replication in independent and more representative samples. Longitudinal research is also needed to establish temporal stability, sensitivity to change, comparability across professional groups, and associations with actual migration behavior.

Future confirmatory studies could test whether Migration Mooring Barriers moderate the WPPF → MR or IMB → MR relationships, using prespecified interaction hypotheses and independently validated measures.

Overall, the study supports the relevance of the Push–Pull–Mooring framework for examining factors associated with pharmacists’ Migration Readiness. The selected model displayed internal cross-validated predictive performance for questionnaire responses, but it has not been validated as an individual prediction, workforce-monitoring, or decision-support instrument. Further longitudinal research, independent-sample replication, measurement-invariance testing, and evidence linking Migration Readiness to subsequent migration behavior are required before any operational application can be considered.

## Figures and Tables

**Figure 1:**
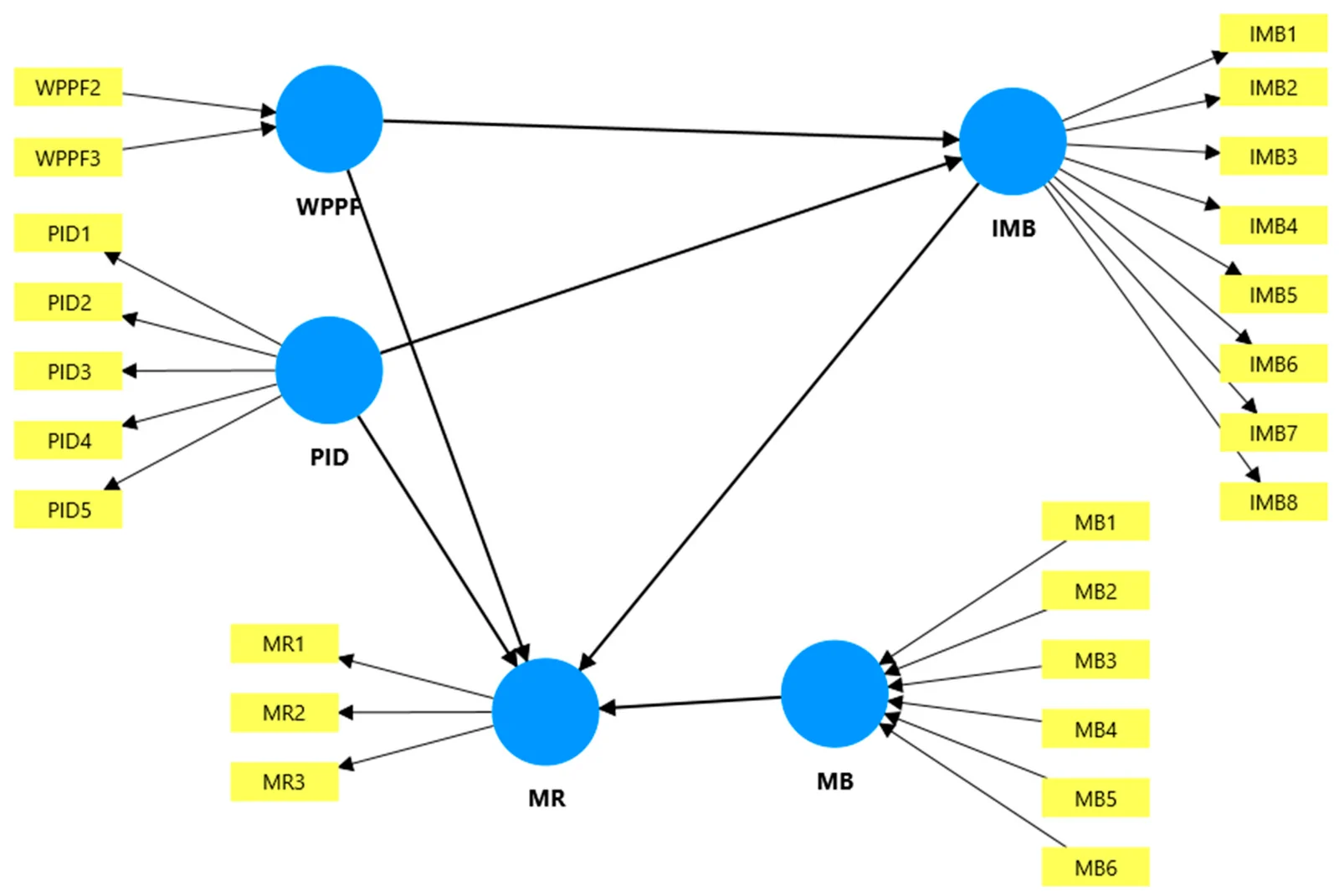
The proposed structural model.

**Figure 2:**
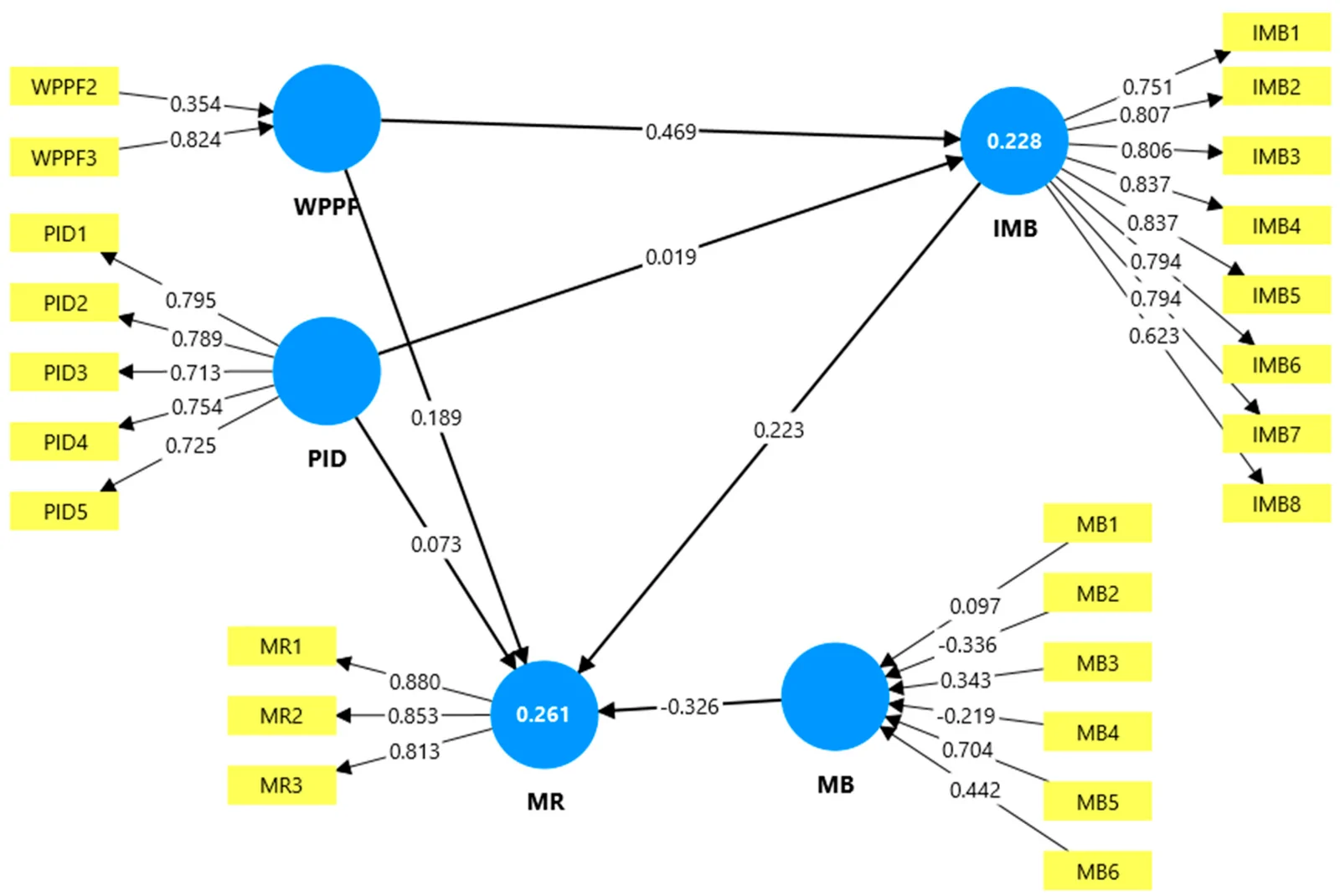
The estimated structural model in SmartPLS.

**Table 1:** Socio-demographic characteristics of the respondents.

Variable	Category	No. of Respondents	% of Total
Gender	Female	485	89.48%
	Male	57	10.52%
Area of residence	Rural	79	14.58%
	Urban	463	85.42%
Education level	PhD	36	6.64%
	Bachelor’s degree in Pharmacy	312	57.56%
	Master’s/Postgraduate studies	194	35.79%
Professional experience	<1 year	22	4.06%
	1–3 years	56	10.33%
	4–10 years	180	33.21%
	>10 years	284	52.40%
Main field of activity	Other field	25	4.61%
	Research/Academia	28	5.17%
	Pharmaceutical distribution/warehouse	24	4.43%
	Community pharmacy	372	68.63%
	Hospital pharmacy	58	10.70%
	Pharmaceutical industry	35	6.46%
Professional status	Employee	425	78.41%
	Self-employed/Collaboration contract	19	3.51%
	Manager/Coordinator	98	18.08%

Note: Percentages describe the obtained convenience sample and should not be interpreted as estimates of the demographic or professional distribution of the Romanian pharmacist population.

**Table 2:** Model constructs, indicators and psychometric properties.

Indicator	Meaning of the Indicator	Cronbach’s Alpha	rho_A	rho_C	AVE
Work and Pay Push Factors—WPPF. Construct developed by the authors based on [3,4,5,7]					
WPPF2	Dissatisfaction with current working conditions	–	–	–	–
WPPF3	Dissatisfaction with the current level of remuneration				
Perceived Institutional and Professional Deficit—PID. Construct adapted from [3,4,20,23]					
PID1	Perceived insufficiency of support from public institutions and competent authorities	0.814	0.827	0.869	0.571
PID2	Perceived insufficiency of societal recognition and appreciation of pharmacists				
PID3	Perceived insufficiency of professional development opportunities				
PID4	Perceived insufficiency of support from professional organizations				
PID5	Perceived lack of favorable professional development and advancement prospects				
Importance of Migration Benefits—IMB. Construct adapted from [1,3,4]					
IMB1	Importance of a higher salary when deciding whether to work abroad	0.909	0.915	0.927	0.614
IMB2	Importance of better working conditions				
IMB3	Importance of professional development and specialization opportunities				
IMB4	Importance of career stability and security				
IMB5	Importance of greater professional recognition and social respect				
IMB6	Importance of a better standard of living for the family				
IMB7	Importance of a more organized and predictable professional environment				
IMB8	Importance of international professional collaborations and experiences				
Migration Mooring Barriers—MB. Construct developed by the authors based on [3,4,13,14,30]					
MB1	Lack of professional-level foreign-language proficiency	–	–	–	
MB2	Procedures for recognizing diplomas and professional qualifications				
MB3	Relocation and adaptation costs				
MB4	Difficulty obtaining information about the employment process				
MB5	Family ties, children, and responsibilities in Romania				
MB6	Uncertainty about adapting to another professional system				
Migration Readiness—MR. Construct adapted from [3,4,48]					
MR1	Consideration of working abroad and the presence of concrete preparatory steps	0.807	0.810	0.886	0.721
MR2	Intended time horizon for a possible departure				
MR3	Assessment of migration as a realistic career alternative				

**Table 3:** Evaluation of formative constructs.

Construct/Indicator	VIF	Outer Weight	*t*	*p*	Outer Loading	*p*
WPPF2—Dissatisfaction with working conditions	1.127	0.354	3.796	<0.001	0.631	<0.001
WPPF3—Dissatisfaction with remuneration	1.127	0.824	13.035	<0.001	0.943	<0.001
MB1—Language barrier	1.431	0.097	0.675	0.5	0.321	0.01
MB2—Qualification-recognition procedures	1.931	−0.336	2.063	0.039	0.074	0.573
MB3—Relocation and adaptation costs	1.91	0.343	1.995	0.046	0.436	<0.001
MB4—Difficulty obtaining information about the employment process	2.181	−0.219	1.252	0.21	0.247	0.049
MB5—Family ties and responsibilities	1.173	0.704	6.247	<0.001	0.858	<0.001
MB6—Uncertainty regarding adaptation	1.745	0.442	2.899	0.004	0.665	<0.001

**Table 4:** Collinearity of indicators—VIF for the outer model.

**Indicator**	**WPPF2**	**WPPF3**	**PID1**	**PID2**	**PID3**	**PID4**	**PID5**	**IMB1**	**IMB2**	**IMB3**	**IMB4**	**IMB5**
**VIF**	1.127	1.127	1.609	1.787	1.516	1.908	1.758	2.093	2.581	2.560	3.002	2.807
**Indicator**	**IMB6**	**IMB7**	**IMB8**	**MB1**	**MB2**	**MB3**	**MB4**	**MB5**	**MB6**	**MR1**	**MR2**	**MR3**
**VIF**	2.465	2.197	1.520	1.431	1.931	1.910	2.181	1.173	1.745	2.433	2.276	1.420

**Table 5:** Assessment of discriminant validity.

Construct	Fornell–Larcker: MR	Fornell–Larcker: IMB	Fornell–Larcker: PID	HTMT: MR	HTMT: IMB	HTMT: PID
**MR**	**0.849**					
**IMB**	0.328	**0.784**		0.371		
**PID**	0.251	0.227	**0.756**	0.305	0.253	

**Table 6:** Path Coefficients and Hypothesis Testing.

Hypothesis and Relationship	Coefficient	t	*p*	95% CI	f^2^	Result
H1: WPPF → IMB	0.469	7.542	<0.001	[0.339; 0.584]	0.228	Supported
H2: WPPF → MR	0.189	3.924	<0.001	[0.092; 0.280]	0.032	Supported
H3: PID → IMB	0.019	0.366	0.714	[−0.075; 0.130]	<0.001	Not supported
H4: PID → MR	0.073	1.730	0.084	[−0.008; 0.158]	0.006	Not supported
H5: IMB → MR	0.223	5.539	<0.001	[0.141; 0.300]	0.052	Supported
H6: MB → MR	−0.326	9.024	<0.001	[−0.406; −0.264]	0.140	Supported
H7: WPPF → IMB → MR	0.105	4.609	<0.001	[0.062; 0.151]	—	Supported
H8: PID → IMB → MR	0.004	0.363	0.716	[−0.016; 0.031]	—	Not supported

**Table 7:** Cross-tabulation of MR1 and MR2 response categories.

MR1\MR2	1	2	4	5	Total
1	182	1	0	0	183
2	54	27	8	3	92
4	32	68	103	15	218
5	3	1	9	36	49
Total	271	97	120	54	542

**Table 8:** Complementary ordinal logistic regression models for MR1 and MR2.

Predictor	MR1 b	MR1 OR	MR1 *p*	MR2 b	MR2 OR	MR2 *p*
WPPF	0.333	1.394	0.002	0.501	1.651	<0.001
PID	0.160	1.173	0.096	0.177	1.194	0.080
IMB	0.400	1.492	<0.001	0.362	1.435	0.004
MB	−0.580	0.560	<0.001	−0.542	0.582	<0.001

Note: Cumulative-logit models with standardized PLS construct scores as predictors. Positive coefficients indicate higher odds of being in a higher-ordered response category. OR = odds ratio.

**Table 9:** PLSpredict results for endogenous construct indicators.

Indicator	Q^2^ Predict	RMSE PLS-SEM	RMSE LM	MAE PLS-SEM	MAE LM
IMB1	0.148	0.788	0.804	0.484	0.490
IMB2	0.113	0.798	0.815	0.493	0.508
IMB3	0.088	0.879	0.890	0.612	0.620
IMB4	0.139	0.876	0.891	0.622	0.633
IMB5	0.171	0.712	0.725	0.394	0.409
IMB6	0.135	0.830	0.846	0.514	0.527
IMB7	0.143	0.762	0.762	0.522	0.512
IMB8	0.073	1.138	1.145	0.946	0.939
MR1	0.129	1.392	1.407	1.228	1.249
MR2	0.133	1.391	1.404	1.186	1.197
MR3	0.151	1.194	1.205	0.978	0.983

Note: PLSpredict used 10-fold cross-validation with 10 repetitions. Indicator assessment and final model specification preceded cross-validation. The results therefore represent internal cross-validated predictions from the selected model and not external validation of the complete model-development procedure.

**Table 10:** CVPAT results at the level of endogenous constructs.

Construct	Benchmark	PLS-SEM Loss	Benchmark Loss	Mean Difference	t	*p*
IMB	Indicator mean	0.734	0.834	−0.100	2.811	0.005
MR	Indicator mean	1.766	2.045	−0.279	5.386	<0.001
IMB	Linear model	0.734	0.754	−0.020	2.611	0.009
MR	Linear model	1.766	1.801	−0.035	3.151	0.002
Overall model	Indicator mean	1.015	1.164	−0.149	4.705	<0.001
Overall model	Linear model	1.015	1.039	−0.024	3.767	<0.001

Note: Negative mean loss differences indicate lower predictive loss for PLS-SEM. CVPAT evaluates predictions of held-out questionnaire responses within the cross-validation procedure and does not establish prediction of subsequent migration behavior.

## Data Availability

The data presented in this study are available on request from the corresponding authors. The data are not publicly available due to privacy and ethical restrictions.

## Supplementary Materials

The following supporting information can be downloaded at: https://www.mdpi.com/article/10.3390/healthcare14193204/s1, File S1: Questionnaire—Migration of healthcare professionals; Table S1: Initial and revised operationalization of the measurement model; Table S2: Sensitivity analysis excluding MR2 from Migration Readiness; Table S3: Outer loadings and bootstrap inference for all measurement indicators in the primary model.

## Abbreviations

The following abbreviations are used in this manuscript:

AVE	Average Variance Extracted
CI	Confidence Interval
CVPAT	Cross-Validated Predictive Ability Test
GDPR	General Data Protection Regulation
HTMT	Heterotrait–Monotrait Ratio
LM	Linear Model
MAE	Mean Absolute Error
MB	Migration Mooring Barriers
MR	Migration Readiness
IMB	Importance of Migration Benefits
PID	Perceived Institutional and Professional Deficit
PLS-SEM	Partial Least Squares Structural Equation Modeling
PPM	Push–Pull–Mooring
Q^2^ predict	Predictive Relevance Statistic
R^2^	Coefficient of Determination
RMSE	Root Mean Square Error
SRMR	Standardized Root Mean Square Residual
VIF	Variance Inflation Factor
WPPF	Work and Pay Push Factors
