# Supplementary material for: Exploring Romanian Pharmacists’ Migration Readiness Through the Push–Pull–Mooring Framework: A PLS-SEM Study
Source: Healthcare (Basel). 2026 Sep 28;14(19):3204. doi: 10.3390/healthcare14193204 (PMC13665060; doi:10.3390/healthcare14193204)
Supplement: Supplementary file 1 [file healthcare-14-03204-s001.zip › healthcare-4532770 - File S1.pdf]

RESEARCH

This research is conducted by academic staff of the "Grigore T. Popa" University of Medicine and Pharmacy, Faculty of Pharmacy, Iași, under the coordination of Professor Cătălina Stan, PhD. We aim to identify the intentions, motivations, and barriers related to professional migration (going abroad to work as a pharmacist) among professionally active pharmacists in Romania.

Participation is voluntary, and responses are confidential and anonymous. Completing the questionnaire takes approximately 8–12 minutes and does not involve psychological or social discomfort. By completing the questionnaire, you confirm that you have understood its purpose and agree to participate. All information you provide is confidential and will be used solely for research purposes. The study results will be presented at scientific events or in published papers, without disclosing personal data that could allow an individual to be identified by name.

Based on the Universal Declaration of Human Rights, Government Ordinance No. 57/2002 on scientific research and technological development, approved by Law No. 324/2003, as well as the Code of Ethics of the pharmacy profession, as a study participant you have the right to refuse participation in the study and the right to request explanations and clarifications regarding the aims, procedures, or results of the study in which you participate.

* Indicates a required question

If you agree to participate in the research, please sign below.

☐ I agree

QUESTIONNAIRE – MIGRATION OF HEALTHCARE PROFESSIONALS (ACTIVE PHARMACISTS)

1. Sex:
☐ Female
☐ Male
2. Age: ____ years
3. Place of residence:
☐ Urban
☐ Rural
4. Education level:
☐ Bachelor’s degree in Pharmacy
☐ Master’s degree / Postgraduate studies
☐ PhD
5. Years of professional experience:
☐ < 1 year
☐ 1–3 years
☐ 4–10 years
☐ >10 years
6. Main field of activity:
☐ Community pharmacy
☐ Hospital pharmacy
☐ Pharmaceutical industry
☐ Pharmaceutical distribution / warehouse
☐ Research / teaching
☐ Other field: ___________________
7. Professional status:
☐ Employee
☐ Manager / coordinator
☐ Self-employed / collaboration contract
8. Do you have relatives or close friends working abroad in the pharmaceutical field?
☐ Yes
☐ No
9. What measures would make you stay and practice in Romania? (select a maximum of 3 options)
☐ Salary increases
☐ Reduction of bureaucracy
☐ Recognition and payment of pharmaceutical services
☐ Access to continuing education and specialization programs
☐ Creation of a modern and fair working environment
☐ Incentives for pharmacists in rural areas
☐ Professional recognition and respect

☐ Greater decision-making autonomy
☐ Flexible working hours
☐ Measures to improve work–life balance
☐ Other suggestions: ___________________________

10. Have you ever considered working abroad as a pharmacist?
☐ Yes, I have already taken concrete steps
☐ Yes, but only as a future possibility
☐ I am not sure
☐ I do not intend to

11. If yes, within what time frame would you like to leave?
☐ Within the next 12 months
☐ Within the next 2–3 years
☐ After 4–5 years
☐ I do not intend to leave

12. Have you ever received a job offer from abroad?
☐ Yes, and I accepted it / worked temporarily
☐ Yes, but I did not accept it
☐ No

13. If I had equivalent professional opportunities in Romania, I would not leave?
☐ Yes
☐ No

14. Preferred country for a possible relocation:
☐ Germany
☐ France
☐ United Kingdom
☐ USA
☐ Other countries: ____________________

Instruction: Rate on a scale from 1 to 5 how important each reason is to you when making the decision to leave abroad as a pharmacist (1 = not at all important, 2 = slightly important, 3 = important, 4 = quite important, 5 = very important).

No.	1	2	3	4	5
**15. Higher salary**					
**16. Better working conditions (equipment, schedule, sufficient staff)**					
**17. Professional development and specialization**					
**18. Career stability and security**					
**19. Greater professional recognition and social respect**					
**20. Better standard of living for the family**					
**21. More organized and predictable environment**					
**22. International professional collaborations and experiences**					
**23. Disappointment with the healthcare system in**					

**Romania**					

Rate on a scale from 1 to 5 the difficulties you perceive that could prevent you from leaving abroad as a pharmacist (1 = not a difficulty, 2 = a minor difficulty, 3 = a difficulty, 4 = a major difficulty, 5 = a very major difficulty).

**No.**	**1**	**2**	**3**	**4**	**5**
**24. Lack of professional-level foreign language proficiency**					
**25. Procedures for recognition of the diploma and qualification**					
**26. Relocation and adaptation costs**					
**27. Difficulty obtaining information about the employment process**					
**28. Family ties / children / responsibilities in Romania**					
**29. Uncertainty about adapting to another professional system**					

30. Are you satisfied with your current working conditions?
☐ Very dissatisfied
☐ Dissatisfied
☐ Neither satisfied nor dissatisfied
☐ Satisfied
☐ Very satisfied
31. The current salary level is:
☐ Unsatisfactory
☐ Partially satisfactory
☐ Satisfactory
☐ Very satisfactory
32. How do you assess the level of support provided to the pharmacy profession by public institutions and competent authorities?
☐ Very low
☐ Low
☐ Medium
☐ High
33. I consider that the pharmacy profession is sufficiently valued in society.
☐ Not at all
☐ To a small extent
☐ To some extent
☐ To a great extent
☐ To a very great extent
34. I consider that the role and contribution of pharmacists are sufficiently recognized and appreciated in society.
☐ Not at all
☐ To a small extent

☐ To some extent
☐ To a great extent
☐ To a very great extent

35. I consider that the professional development opportunities available to pharmacists are sufficient.

☐ Not at all
☐ To a small extent
☐ To some extent
☐ To a great extent
☐ To a very great extent

36. The healthcare system in Romania provides adequate conditions for pharmacists.

☐ Not at all
☐ To a small extent
☐ To some extent
☐ To a great extent
☐ To a very great extent

37. I consider that professional organizations provide sufficient support for the representation and development of the pharmacy profession.

☐ Strongly disagree
☐ Disagree
☐ Neutral
☐ Agree
☐ Strongly agree

38. I consider that pharmacists in Romania have favorable prospects for professional development and advancement.

☐ Strongly disagree
☐ Disagree
☐ Neutral
☐ Agree
☐ Strongly agree

39. Is migration a realistic solution for improving my career?

☐ Strongly disagree
☐ Disagree
☐ Neutral
☐ Agree
☐ Strongly agree

Respond to each statement on a scale from 1 to 5 (1 = strongly disagree, 2 = disagree, 3 = neutral, 4 = agree, 5 = strongly agree).

**No.**	**1**	**2**	**3**	**4**	**5**
**40. The state should implement concrete policies for retaining pharmacists**					
**41. I intend to build a long-term career in Romania**					

Describe how each statement characterizes you now, at the moment you are completing the questionnaire. Using the scale from 1 to 5, indicate your response for each item (1 = not at all characteristic, 2 = slightly characteristic, 3 = neutral, 4 = characteristic, 5 = very characteristic).

**No.**	**1**	**2**	**3**	**4**	**5**
**42. I am the life of the party**					
**43. I do not talk a lot**					
**44. I talk to many different people at parties**					
**45. I keep in the background**					
**46. I feel other people's emotions**					
**47. I am not interested in other people's problems**					
**48. I have a sensitive heart**					
**49. I insult people**					
**50. I am always prepared**					
**51. I leave my belongings lying around**					
**52. I pay attention to details**					
**53. I make a mess**					
**54. I get stressed easily**					
**55. I am relaxed most of the time**					
**56. I worry**					
**57. I rarely feel sad**					
**58. I have a rich vocabulary**					
**59. I have difficulty understanding abstract ideas**					
**60. I have a rich imagination**					
**61. I am not interested in abstract ideas**					
