# Supplementary material for: Exploring Romanian Pharmacists’ Migration Readiness Through the Push–Pull–Mooring Framework: A PLS-SEM Study
Source: Healthcare (Basel). 2026 Sep 28;14(19):3204. doi: 10.3390/healthcare14193204 (PMC13665060; doi:10.3390/healthcare14193204)
Supplement: Supplementary file 1 [file healthcare-14-03204-s001.zip › healthcare-4532770-supplementary.pdf]

Table S1. Initial and revised operationalization of the measurement model.

Code	Questionnaire item	Response scale and coding	Initial construct	Revised status
WPPF1/Q23	Disappointment with the healthcare system in Romania	Importance from 1 = not at all important to 5 = very important	WPPF	Excluded because the stem measured importance as a migration motive rather than current dissatisfaction
WPPF2/Q30	Satisfaction with current working conditions	1 = very dissatisfied to 5 = very satisfied; reverse-coded	WPPF	Retained
WPPF3/Q31	Assessment of current salary level	1 = unsatisfactory to 4 = very satisfactory; reverse-coded	WPPF	Retained
PID1/Q32	Support provided to the pharmacy profession by public institutions and competent authorities	Four ordered categories from very low to high; reverse-coded	PID	Retained
PID2/Q34	Recognition and appreciation of pharmacists' role and contribution	1 = not at all to 5 = to a very great extent; reverse-coded	PID	Retained
PID3/Q35	Sufficiency of professional development opportunities	1 = not at all to 5 = to a very great extent; reverse-coded	PID	Retained
PID4/Q37	Support from professional organizations for representation and development	1 = strongly disagree to 5 = strongly agree; reverse-coded	PID	Retained
PID5/Q38	Favorable prospects for professional development and advancement	1 = strongly disagree to 5 = strongly agree; reverse-coded	PID	Retained
IMB1/Q15	Higher salary	Importance from 1 = not at all important to 5 = very important	IMB	Retained
IMB2/Q16	Better working conditions	Same five-category importance scale	IMB	Retained
IMB3/Q17	Professional development and specialization	Same five-category importance scale	IMB	Retained
IMB4/Q18	Career stability and security	Same five-category importance scale	IMB	Retained
IMB5/Q19	Greater professional recognition and social respect	Same five-category importance scale	IMB	Retained
IMB6/Q20	Better standard of living for the family	Same five-category importance scale	IMB	Retained
IMB7/Q21	More organized and predictable environment	Same five-category importance scale	IMB	Retained
IMB8/Q22	International professional collaborations and experiences	Same five-category importance scale	IMB	Retained
MB1/Q24	Lack of professional-level foreign-language proficiency	Difficulty from 1 = not a difficulty to 5 = a very major difficulty	MB	Retained
MB2/Q25	Procedures for recognition of the diploma and qualification	Same five-category difficulty scale	MB	Retained

MB3/Q26	Relocation and adaptation costs	Same five-category difficulty scale	MB	Retained
MB4/Q27	Difficulty obtaining information about the employment process	Same five-category difficulty scale	MB	Retained
MB5/Q28	Family ties, children, and responsibilities in Romania	Same five-category difficulty scale	MB	Retained
MB6/Q29	Uncertainty about adapting to another professional system	Same five-category difficulty scale	MB	Retained
MR1/Q10	Consideration of working abroad and concrete preparatory steps	Four ordered response categories, recoded so that higher values indicate greater readiness	MR	Retained
MR2/Q11	Intended time horizon for a possible departure	Four ordered response categories, recoded so that higher values indicate greater temporal proximity	MR	Retained in the primary model; excluded in the sensitivity model
MR3/Q39	Migration as a realistic solution for career improvement	1 = strongly disagree to 5 = strongly agree	MR	Retained

Note: Higher analytical values indicate higher levels of the corresponding construct. Positively worded satisfaction, institutional-support, recognition, and professional-development indicators were reverse-coded when used to measure dissatisfaction or perceived deficit. MR1 and MR2 were treated as ordered variables and coded so that higher categories represented greater migration readiness.

Table S2. Sensitivity analysis excluding MR2 from Migration Readiness.

Relationship	Primary model β	t	p	95% CI	Model without MR2 β	t	p	95% CI	Robustness conclusion
WPPF → IMB	0.469	7.542	<0.001	[0.339, 0.584]	0.469	7.531	<0.001	[0.338, 0.584]	Stable; supported in both models
WPPF → MR	0.189	3.924	<0.001	[0.092, 0.280]	0.177	3.326	<0.001	[0.070, 0.277]	Stable; supported in both models
PID → IMB	0.019	0.366	0.714	[−0.075, 0.130]	0.019	0.359	0.72	[−0.075, 0.130]	Stable; not supported in either model
PID → MR	0.073	1.73	0.084	[−0.008, 0.158]	0.053	1.231	0.218	[−0.029, 0.142]	Stable; not supported in either model
IMB → MR	0.223	5.539	<0.001	[0.141, 0.300]	0.263	6.21	<0.001	[0.177, 0.342]	Stable; supported in both models
MB → MR	−0.326	9.024	<0.001	[−0.406, −0.264]	−0.317	8.88	<0.001	[−0.395, −0.256]	Stable; supported in both models
WPPF → IMB → MR	0.105	4.609	<0.001	[0.062, 0.151]	0.123	4.918	<0.001	[0.076, 0.174]	Stable; supported in both models
PID → IMB → MR	0.004	0.363	0.716	[−0.016, 0.031]	0.005	0.357	0.721	[−0.019, 0.036]	Stable; not supported in either model

Panel B. Explained variance and model fit.

Statistic	Primary model	Model without MR2	Robustness conclusion
R^2 IMB	0.228	0.228	Essentially unchanged
Adjusted R^2 IMB	0.225	0.225	Essentially unchanged
R^2 MR	0.261	0.266	Slight increase; substantive conclusion unchanged
Adjusted R^2 MR	0.256	0.261	Slight increase; substantive conclusion unchanged
Estimated-model SRMR	0.056	0.055	Essentially unchanged

Note: The primary model included MR1, MR2, and MR3 as reflective indicators of Migration Readiness. The sensitivity model was identical to the primary model except that MR2 was excluded, leaving MR1 and MR3 as indicators of Migration Readiness. Both models retained all six formative indicators of Migration Mooring Barriers. β = standardized path coefficient; CI = confidence interval; IMB = Importance of Migration Benefits; MB = Migration Mooring Barriers; MR = Migration Readiness; PID = Perceived Institutional and Professional Deficit; WPPF = Work and Pay Push Factors. Statistical inference was based on bootstrapping with 10,000 subsamples, percentile 95% confidence intervals, and two-tailed tests. "Stable" indicates that the direction, statistical significance, and substantive interpretation of the relationship remained unchanged after excluding MR2. SRMR refers to the estimated model.

Table S3. Outer loadings and bootstrap inference for all measurement indicators in the primary model.

Indicator	Measurement type	Outer loading	t	p	95% percentile CI
IMB1	Reflective	0.751	18.829	<0.001	[0.663; 0.819]
IMB2	Reflective	0.807	24.43	<0.001	[0.733; 0.861]
IMB3	Reflective	0.806	28.195	<0.001	[0.743; 0.855]
IMB4	Reflective	0.837	40.456	<0.001	[0.791; 0.872]
IMB5	Reflective	0.837	30.037	<0.001	[0.774; 0.883]
IMB6	Reflective	0.794	26.057	<0.001	[0.727; 0.846]
IMB7	Reflective	0.794	23.329	<0.001	[0.716; 0.851]
IMB8	Reflective	0.623	16.858	<0.001	[0.544; 0.690]
PID1	Reflective	0.795	24.896	<0.001	[0.723; 0.849]
PID2	Reflective	0.789	26.361	<0.001	[0.724; 0.841]
PID3	Reflective	0.713	18.314	<0.001	[0.629; 0.779]
PID4	Reflective	0.754	17.152	<0.001	[0.655; 0.824]
PID5	Reflective	0.725	14.786	<0.001	[0.610; 0.802]
MR1	Reflective	0.88	72.173	<0.001	[0.855; 0.903]
MR2	Reflective	0.853	54.859	<0.001	[0.821; 0.882]
MR3	Reflective	0.813	43.528	<0.001	[0.771; 0.844]
WPPF2	Formative	0.631	8.676	<0.001	[0.485; 0.773]
WPPF3	Formative	0.943	27.9	<0.001	[0.855; 0.985]
MB1	Formative	0.321	2.583	0.01	[0.059; 0.544]
MB2	Formative	0.074	0.564	0.573	[−0.190; 0.323]
MB3	Formative	0.436	3.636	<0.001	[0.171; 0.646]
MB4	Formative	0.247	1.968	0.049	[−0.019; 0.473]
MB5	Formative	0.858	11.446	<0.001	[0.655; 0.945]
MB6	Formative	0.665	6.793	<0.001	[0.432; 0.812]

Note: For reflective constructs, arrows run from the construct to the indicators. For formative composites, the reported loadings represent absolute indicator contributions and complement the relative outer weights reported in Table 3. Confidence intervals are percentile bootstrap intervals based on 10,000 subsamples.
